# Supplementary material for: Sub-populations of Spinal V3 Interneurons Form Focal Modules of Layered Pre-motor Microcircuits
Source: Cell Rep. 2018 Oct 2;25(1):146–156.e3. doi: 10.1016/j.celrep.2018.08.095 (PMC6180347; doi:10.1016/j.celrep.2018.08.095)
Supplement: Document S1. Figures S1–S3 [file mmc1.pdf]

Cell Reports, Volume *25*

Supplemental Information

Sub-populations of Spinal V3 Interneurons Form

Focal Modules of Layered Pre-motor Microcircuits

Jeremy W. Chopek, Filipe Nascimento, Marco Beato, Robert M. Brownstone, and Ying Zhang

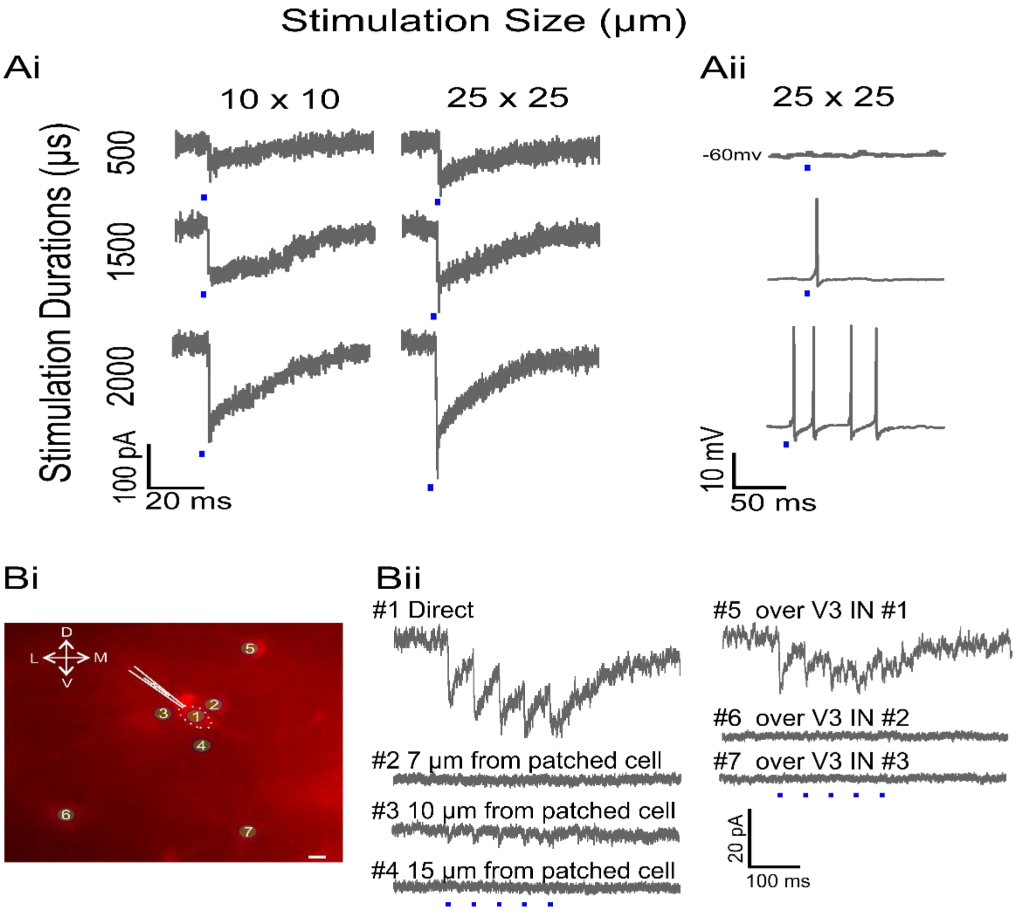

Figure S1. Sensitivity and specificity of photostimulation. Related to Figure 1.

A. Sensitivity of photostimulation. (Ai) Voltage clamp responses to different photostimulation areas (10 x 10 μm and 25 x 25 μm squares) and durations (1000, 1500, 2000 μs) directly over a patched motoneuron. (Aii) Current clamp responses to different durations of photostimulation. **B.** Specificity of photostimulation. (Bi) Spots for photostimulation were placed over the patched V3 IN soma (1), near the patched neuron (2, 3, 4) and on three identifiable pre-synaptic V3 IN somata (5, 6, 7). Scale bar = 10 μm. (Bii) Voltage-clamp recordings showing that the recorded V3 IN responded to direct but not adjacent stimulation as well as to stimulation of 1 of the 3 other V3 INs, demonstrating specificity of stimulation.

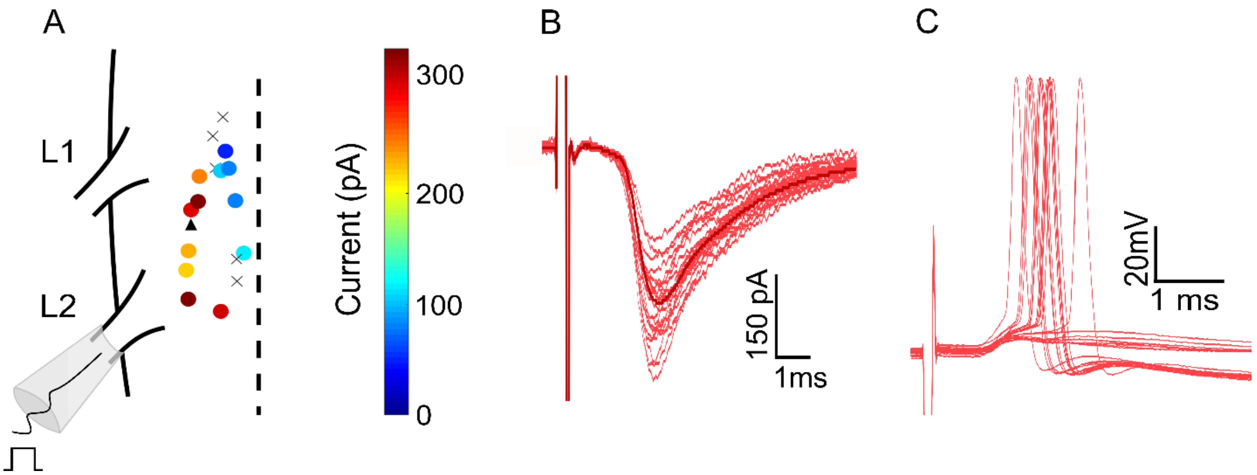

Figure S2. Recurrent excitation of V3 INs by motoneurons in the dorsal horn removed preparation. Related to Figure 4.

(A) Dorsal horn removed preparation in which a suction electrode was used to stimulate the L2 ventral root while recording V3 INs. V3 IN locations are depicted as circles (responded to ventral root stimulation) and X's (did not respond to ventral root stimulation) with colour indicating the strength of the evoked EPSC. Dashed line indicates the central canal. Black arrowhead indicates the location of the V3 IN described in panel B & C. (B) Individual voltage-clamp responses recorded in a patched $V3_{VLat}$ IN in response to L2 ventral root stimulation (30 pulses). Note stimulus artefact has been truncated and subtracted (by fitting with a double exponential). Thick red line indicates average evoked response. (C) Individual current-clamp responses recorded in the same patched V3Vlat IN as in (B) in response to L2 ventral root stimulation.

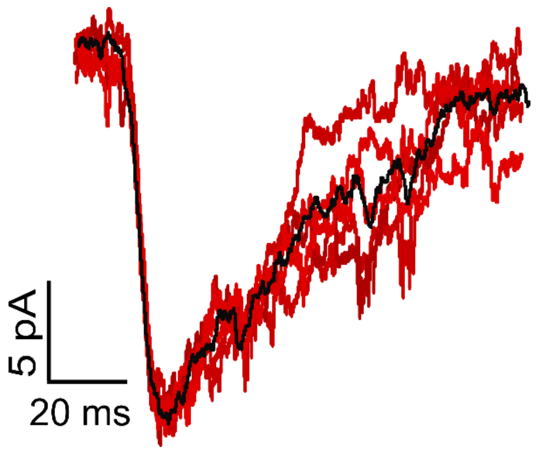

Figure S3. Low jitter of V3$_{VMed}$ IN-evoked EPSCs in V3$_{VLat}$ INs. Related to Figure 5. Responses in a V3$_{VLat}$ IN to 5 consecutive stimulation pulses at 1Hz to a V3$_{VMed}$ IN (red shades), with the average overlaid (black).
