# Supplementary material for: Sub-populations of Spinal V3 Interneurons Form Focal Modules of Layered Pre-motor Microcircuits
Source: Cell Rep. 2018 Oct 2;25(1):146–156.e3. doi: 10.1016/j.celrep.2018.08.095 (PMC6180347; doi:10.1016/j.celrep.2018.08.095)
Supplement: Document S2. Article plus Supplemental Information [file mmc2.pdf]

Cell Reports

Article

Sub-populations of Spinal V3 Interneurons Form Focal Modules of Layered Pre-motor Microcircuits

Graphical Abstract

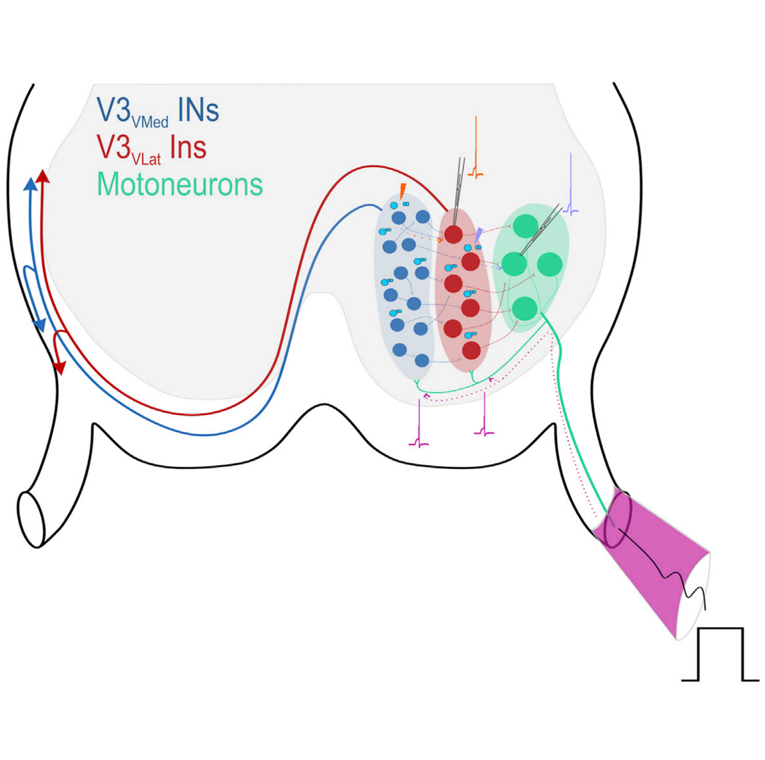

Authors

Jeremy W. Chopek, Filipe Nascimento, Marco Beato, Robert M. Brownstone, Ying Zhang

Correspondence

r.brownstone@ucl.ac.uk (R.M.B.),
ying.zhang@dal.ca (Y.Z.)

In Brief

Using electrophysiology combined with holographic photostimulation, Chopek et al. demonstrate focal layered microcircuits within the spinal cord. These microcircuits are composed of two ventral V3 interneuron sub-populations and ipsilateral motoneurons. Synaptic connectivity was established from medial to lateral, with motoneurons recurrently exciting both V3 interneuron sub-populations.

Highlights

- Two populations of ventral spinal V3 interneurons (INs) can be distinguished
- Medial ($V3_{VMed}$) and lateral ($V3_{VLat}$) populations differ in connectivity patterns
- Motoneuron axons recurrently excite ipsilateral V3 INs
- Ventral spinal V3 INs form layered microcircuits for motor output

Chopek et al., 2018, Cell Reports *25*, 146–156
October 2, 2018 © 2018 The Author(s).
https://doi.org/10.1016/j.celrep.2018.08.095

CellPress

OPEN ACCESS
CellPress

Cell Reports
Article

Sub-populations of Spinal V3 Interneurons Form Focal Modules of Layered Pre-motor Microcircuits

Jeremy W. Chopek,[1,2] Filipe Nascimento,[3] Marco Beato,[3] Robert M. Brownstone,[2,4,*] and Ying Zhang[1,*]
[1]Department of Medical Neuroscience, Faculty of Medicine, Dalhousie University, Halifax, NS B3H 4R2, Canada
[2]Sobell Department of Neuromuscular Diseases, Institute of Neurology, University College London, London WC1N 3BG, UK
[3]Department of Neuroscience, Physiology and Pharmacology, University College London, London WC1E 6BT, UK
[4]Lead Contact
*Correspondence: r.brownstone@ucl.ac.uk (R.M.B.), ying.zhang@dal.ca (Y.Z.)
https://doi.org/10.1016/j.celrep.2018.08.095

SUMMARY

Layering of neural circuits facilitates the separation of neurons with high spatial sensitivity from those that play integrative temporal roles. Although anatomical layers are readily identifiable in the brain, layering is not structurally obvious in the spinal cord. But computational studies of motor behaviors have led to the concept of layered processing in the spinal cord. It has been postulated that spinal V3 interneurons (INs) play multiple roles in locomotion, leading us to investigate whether they form layered microcircuits. Using patch-clamp recordings in combination with holographic glutamate uncaging, we demonstrate focal, layered modules, in which ventromedial V3 INs form synapses with one another and with ventrolateral V3 INs, which in turn form synapses with ipsilateral motoneurons. Motoneurons, in turn, provide recurrent excitatory, glutamatergic input to V3 INs. Thus, ventral V3 interneurons form layered microcircuits that could function to ensure well-timed, spatially specific movements.

INTRODUCTION

Over the course of evolution, nervous systems developed neuronal layers for information processing. This is perhaps most evident in the primate cerebral and cerebellar cortices, in which structural layers can be beautifully visualized. Although these structural layers likely evolved for geometric reasons, in particular for economies of wiring (Shipp, 2007), functional layers appeared even in the absence of structure. For example, complex behaviors can be produced by layered stages of processing in bird brains, in the absence of a laminated architecture (Calabrese and Woolley, 2015). Thus in the pallium, layered processing, which likely confers staged processing capabilities, appeared hundreds of millions of years ago, much earlier than structural layers (Calabrese and Woolley, 2015).

The spinal cord predates the pallium, and models of spinal circuits for locomotion routinely implicate a layered organization as being necessary for the flexibility of motor rhythms and patterns (e.g., Danner et al., 2017; Lafreniere-Roula and McCrea, 2005). But evidence that supports layered processing in the spinal cord has been indirect, and results largely from studies of "errors" in stepping called deletions (Dyck et al., 2012; Griener et al., 2017; Rybak et al., 2006; Zhong et al., 2012). Another approach to examine layered processing in the spinal cord has been to quantify the changes in locomotor behavior when whole cardinal classes of genetically defined neurons are silenced or eliminated from spinal motor circuits (Andersson et al., 2012; Caldeira et al., 2017; Crone et al., 2008, 2009; Dougherty et al., 2013; Enjin et al., 2017; Gosgnach et al., 2006; Lanuza et al., 2004; Talpalar et al., 2013; Zhang et al., 2008, 2014). With these approaches, specific interneuronal classes have been assigned to different layers of processing: a rhythm-generating layer that projects to a pattern formation layer that in turn forms synaptic connections with motoneurons (Dyck et al., 2012; Griener et al., 2017; Zhong et al., 2012). From these studies, computational models have been built to support oligosynaptic, layered connectivity diagrams (Danner et al., 2017).

To move from modeling studies to circuit function, it is first necessary to be able to identify specific neurons in the spinal cord despite the apparent lack of structural conformation. Genetic techniques in the mouse have led to the identification of a number of different interneuronal cardinal classes arising from the developing spinal cord (Jessell, 2000). However, most genetically defined interneuronal populations do not form homogeneous populations (Bikoff et al., 2016; Kiehn, 2016). Thus, techniques that manipulate cardinal IN classes demonstrate neither connectivity nor the underlying functional layers involved in movement production. Techniques focusing on functional connectivity between individually identified neurons are therefore needed to define layers of microcircuits involved in movement.

V3 interneurons (INs) develop from the ventral most progenitor domain, p3, in the spinal cord. These excitatory commissural INs are important for ensuring a stable locomotor output (Zhang et al., 2008). Modeling studies suggest that V3 INs play a pivotal role in spinal locomotor circuits, forming direct connections with contralateral rhythm-generating circuits, and playing key roles in gait transitions (Danner et al., 2017; Rybak et al., 2006). The involvement of V3 INs in rhythm generation and the modeling studies together suggest that these neurons are synaptically removed from motoneurons (MNs). Yet anatomical data have demonstrated boutons from V3 INs in apposition to MNs (Zhang et al., 2008). Furthermore, during development, V3 INs diverge

 © 2018 The Author(s).
This is an open access article under the CC BY license (http://creativecommons.org/licenses/by/4.0/).

into several sub-populations designated as dorsal and ventral V3 INs, determined by their location, morphology, and electrophysiological properties (Borowska et al., 2013, 2015). This divergence into sub-populations together with the disparate indicators of V3 IN roles and connectivity suggest that there are different roles for V3 IN sub-populations and led us to ask whether V3 INs form functional layers in the ventral spinal cord.

Using whole-cell patch-clamp recordings in combination with holographic glutamate uncaging, we demonstrate focal layers of neuronal connectivity between two ventral V3 IN sub-populations and ipsilateral MNs in the lateral motor column. These local synapses follow a rule of connectivity in which smaller medial V3 ventral INs form synapses with larger lateral V3 ventral INs, which in turn form synapses with ipsilateral MNs. Thus, ventral V3 INs form layered modular structures that could enhance information processing needed to ensure flexibility in movement production.

RESULTS

Holographic Photolytic Stimulation of MNs and V3 INs

To determine the holographic stimulation parameters needed to evoke action potentials in MNs and V3 INs, we first photolysed caged glutamate (photostimulated) at the soma of the recorded neuron (Figure S1) during voltage-clamp recordings. Consistent with previous findings (Lutz et al., 2008; Zahid et al., 2010), the amplitudes of the responses were related to both the size (which was standardized to a circle encompassing the entire soma of V3 INs or MNs) and duration of photostimulation. The pulse duration, in our case, had the greatest impact on neuronal responses (Figure S1Ai). The “ideal” pulse duration, defined as the duration that consistently produced a single action potential in current-clamp recordings (Figure S1Aii), was 800–1000 μs for ventral V3 INs ($n = 20$) and 1,000–1,500 μs for MNs ($n = 20$). If longer durations were necessary, the slice was discarded, as it was deemed to be unhealthy. These parameters were used for all subsequent experiments.

Ventrolateral V3 INs Form Synapses with Ipsilateral MNs

As boutons from V3 INs have been found in apposition to MNs (Zhang et al., 2008) and as most V3 INs are commissural (Blacklaws et al., 2015; Zhang et al., 2008), we first sought to characterize synapses between V3 INs and contralateral MNs. In lumbar spinal cord slices isolated from P7–P14 *Sim1*$^{Cre/+}$;*Rosa26*^{tdTomato} or *Sim1*$^{Cre/+}$;*Rosa26*^{tdTomato};*Hb9::eGFP* mice, photostimulating ventral ($n > 110$) or dorsal ($n > 100$) V3 INs did not evoke responses in contralateral GFP-positive or putative MNs ($n = 12$; data not shown). Similarly, in dorsal horn removed lumbar spinal cords, no monosynaptic responses were recorded in contralateral MNs in adjacent segments ($n = 11$; data not shown). We therefore asked whether there could be ipsilateral connectivity and indeed found evoked responses in ipsilateral MNs (ipsi-MNs) in the lateral motor column (limb-innervating) when local ventral (see below) but not dorsal ($n = 40$) V3 INs were activated. We then aimed to define local connections between ventral V3 INs and ipsi-MNs.

In 18 recorded ipsi-MNs, 25 connections were found from photostimulation of 120 ventral V3 INs (Figure 1A). Direct photostimulation of ipsi-MNs resulted in consistent generation of action potentials (Figure 1B). In current-clamp recordings, stimulation of 9 of the 25 V3 INs led to ipsi-MN action potentials, while stimulation of the remaining 16 of 25 V3 INs led to subthreshold responses that had the appearance of excitatory post-synaptic potentials (EPSPs; Figures 1C and 1D). In voltage clamp (at −60 mV), these responses had a mean amplitude of 54 ± 24 pA, compared with the direct responses in ipsi-MNs of 110 ± 60 pA (Figures 1E and 1F; $p = 0.004$). The rise times of the inward currents in ipsi-MNs were similar between V3-stimulated versus directly stimulated ipsi-MNs (6.6 ± 0.8 versus 7.0 ± 0.9 ms, $n = 5$ per, $p = 0.63$). No responses were recorded when the stimulation site was moved to an adjacent off cell target (Figures S1Bi and S1Bii), indicating that the responses were not due to diffusion of uncaged glutamate. Together, these data support that the responses seen were synaptically driven.

To further confirm that these ventral V3-evoked ipsi-MN responses were indeed produced by synaptic transmission, we compared the latencies of responses evoked by direct activation of ipsi-MNs with those evoked by activation of ventral V3 INs (Figures 1E and 1G; $n = 9$). Direct photostimulation of ipsi-MNs resulted in responses with a mean latency of 0.6 ± 0.3 ms. This contrasted with the responses produced in the same ipsi-MNs by photostimulation of ventral V3 INs: these responses had a mean onset latency of 3.7 ± 0.9 ms (Figures 1E and 1G; $p < 0.0001$). Of note, the jitters of the onsets of the ventral V3-evoked excitatory post-synaptic currents (EPSCs) were negligible (variance = 2.9×10^{-7} s^2, $n = 5$; Figure 1H). Taken together, these data confirm that ventral V3 INs form monosynaptic connections with ipsi-MNs.

Mapping of photostimulated ventral V3 INs in relation to the recorded MN revealed a clear distinction in sub-populations of V3 INs with respect to ipsi-MN connectivity. All V3 IN-evoked responses (25 of 25 in 18 ipsi-MNs) were evoked by stimulating the most ventral-lateral of the V3 INs. In 7 of the 18 recorded MNs, responses were evoked by stimulation of 2 different V3 INs in this location (Figures 1C and 1D). The distance between the ipsi-MN and the connected V3 INs was always less than 150 μm (mean 116 ± 37 μm, range 35–144 μm). We then classified two sub-populations of ventral V3 INs, initially on the basis of distance of less than or greater than 150 μm from ipsi-MNs, using the terms “ventral-lateral V3” ($V3_{VLat}$) INs and “ventral-medial V3” ($V3_{VMed}$) INs (noting that these are relative terms). Using this criterion, it became apparent that $V3_{VLat}$ INs were relatively sparse (typically 3–4 neurons per 300-μm-thick slice) and large (335 ± 120 μm^2; $n = 7$) compared with the more abundant (typically 8–10 per slice) and smaller (155 ± 50 μm^2; $n = 9$, $p < 0.002$) $V3_{VMed}$ INs (Figures 1I and 1J). Correspondingly, rheobase was significantly lower in $V3_{VMed}$ INs compared with $V3_{VLat}$ INs (15.7 ± 6.8 versus 54.7 ± 18.3 pA, $n = 9$ and 7, $p < 0.00002$), and input resistance was significantly greater in $V3_{VMed}$ INs compared with $V3_{VLat}$ INs (660 ± 190 versus 290 ± 95 MΩ, $n = 9$ and 7, $p < 0.0006$). Thus, ventral V3 INs are clearly segregated into 2 sub-populations, with only the $V3_{VLat}$ INs forming synaptic connections with local ipsi-MNs.

In another set of experiments, the connectivity between V3 INs and MNs was probed by performing loose cell attached stimulation of V3 INs while recording in whole-cell mode from an MN (Bhumbra et al., 2014). One synaptically connected pair ($V3_{VLat}$

OPEN ACCESS
CellPress

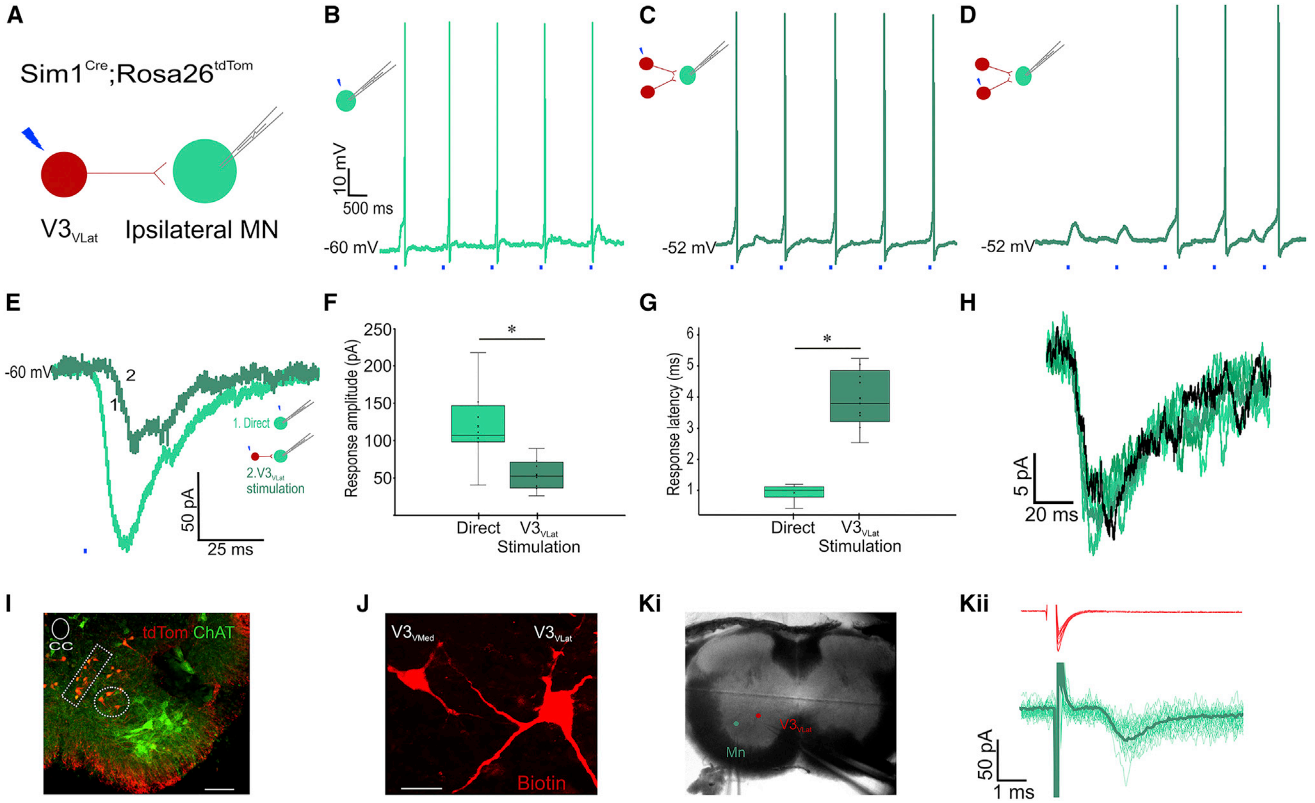

Figure 1. $V3_{VLat}$ INs Excite Ipsilateral MNs

(A) V3 INs were photostimulated while recording from ipsilateral MNs.

(B) Direct photostimulation of patched MN evoked a single action potential per stimulus.

(C and D) Photostimulation of two different $V3_{VLat}$ INs produced action potentials (C) or EPSPs (D) in the same MN.

(E) Voltage-clamp recordings from an MN in response to direct (light green) and $V3_{VLat}$ IN (dark green) photostimulation.

(F and G) Response amplitude was significantly decreased ($n = 9$, $p = 0.004$, Student's t test) (F) and response latency increased ($n = 9$, $p = 0.0001$, Student's t test) (G) following $V3_{VLat}$ IN photostimulation (dark green) compared with direct MN photostimulation (light green). Data shown are mean and quartile range, with circles indicating individual responses.

(H) Responses in an ipsilateral MN to stimulation of a $V3_{VLat}$ IN (five sweeps at 1 Hz; green shades), with the average overlaid (black), demonstrating minimal jitter in response latency.

(I) Transverse section of L2 lumbar spinal cord showing the distribution of tdTom-positive ventral V3 INs (red) and MNs (green). Dashed rectangle and circle refer to the $V3_{VMed}$ and $V3_{VLat}$ IN subpopulations respectively. Scale bar, 100 μm.

(J) A representative example of a $V3_{VMed}$ and $V3_{VLat}$ IN biotin fills. Note the large soma and dendritic branching of the $V3_{VLat}$ compared with the $V3_{VMed}$ IN. Scale bar, 20 μm.

(Ki) Slice showing location of a $V3_{VLat}$ IN (red) that was stimulated in loose-patch configuration while post-synaptic responses were recorded in the whole cell-attached MN (green).

(Kii) Repeated stimulation (30 pulses) of the $V3_{Vlat}$ IN (red, top) produced post-synaptic responses in the recorded MN (green, bottom). Thick green line represents the average response. Note that stimulation artifact was truncated and subtracted (by fitting with a double exponential).

See also Figure S1.

to MN) was found among ∼25 V3 INs tested, mostly selected from the most lateral population (Figures 1Ki and 1Kii). Taken together, these data demonstrate that $V3_{VLat}$ INs form monosynaptic connections with ipsi-MNs.

$V3_{VLat}$ INs that Form Synapses with Ipsi-MNs Also Have Commissural Axons

As studies have demonstrated that V3 INs are primarily commissural, we next asked whether neurons of the ipsilateral-projecting $V3_{VLat}$ IN population also have contralateral projections. To answer this, green fluorescent dextran crystals were applied to a small unilateral incision of the L3 segment spinal cord to retrogradely label contralaterally projecting neurons (Figures 2A and 2B1). After incubation, slices from the L2 segment were prepared, and MNs contralateral to dextran administration were recorded, while dextran-positive V3 INs (ipsilateral to MNs) were photostimulated (Figures 2A and 2B4). Both $V3_{VLat}$ and $V3_{VMed}$ INs were dextran positive, suggesting that both sub-populations have descending commissural projections (Figures 2B2–2B4, arrowheads). Photostimulation of commissural $V3_{VLat}$ INs (3 out of 4 cells) led to local, ipsi-MN responses (Figure 2C). These data demonstrate that $V3_{VLat}$ INs have bifurcating axons,

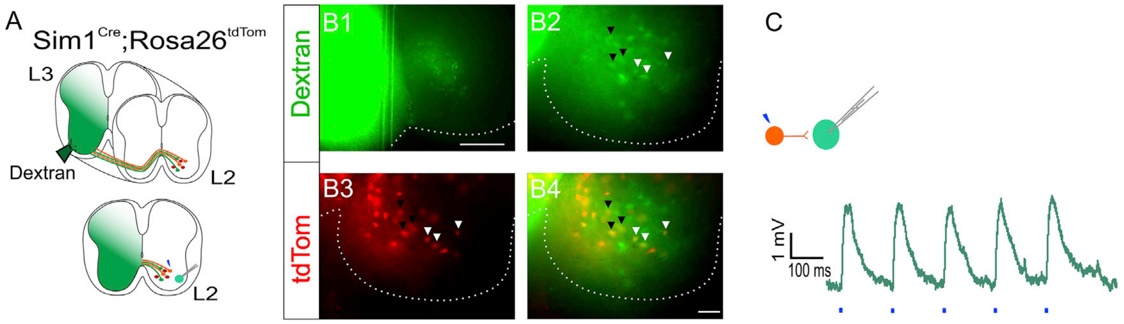

Figure 2. $V3_{VLat}$ INs Project Both Ipsilaterally and Contralaterally in the Spinal Cord

(A) Green fluorescent dextran was applied unilaterally at L3 to label contralateral L2 ventral V3 INs. MNs were then recorded in L2 sections while photostimulating dextran-positive $V3_{VLat}$ INs (orange).

(B) Representative images of dextran labeled spinal cord section used for recordings. (B1) Unilateral dextran application site with contralateral neuronal labeling. Scale bar, 100 μm. (B2) Contralateral dextran-positive neurons. (B3) tdTom-expressing V3 INs (red). (B4) Overlay of (B2) and (B3). White and black arrowheads denote dextran-positive $V3_{VLat}$ INs and $V3_{VMed}$ INs, respectively. White dashed line outlines ventral horn of spinal cord. Scale bar, 20 μm.

(C) Photostimulation of dextran-positive $V3_{VLat}$ INs produced EPSPs in patched MNs.

with an ipsilateral branch forming synapses with local ipsi-MNs and a commissural branch with a descending projection.

Connectivity between $V3_{VLat}$ INs and MNs Is Bidirectional

To confirm that the connections between $V3_{VLat}$ INs and ipsi-MNs are glutamatergic synapses, we recorded from ipsi-MNs in $V3^{OFF}$ mice (*Sim1$^{Cre/+}$; vGluT2$^{flox/flox}$; Rosa26^{tdTom}*), in which expression of vGluT2 was selectively deleted in V3 INs. In L2 spinal cord slices from these mice, EPSPs but not action potentials were evoked in MNs by photostimulation of $V3_{VLat}$ INs (responses in 4 of 5 MNs from 4 of 25 $V3_{VLat}$ INs; Figure 3A). The evoked EPSCs were of small amplitude (14 ± 6 pA, ~75% lower than those in $V3^{ON}$ mice; Figure 3B), with a mean latency of 3.7 ± 0.4ms (similar to that in $V3^{ON}$ mice). The presence of an evoked response suggests that the knockout strategy was either incomplete or that a component of the response was vGluT2 independent.

In characterizing the connectivity between $V3_{VLat}$ INs and ipsi-MNs, we next attempted to simultaneously patch-clamp $V3_{VLat}$ INs and their connected ipsi-MNs in slices from both $V3^{ON}$ (*Sim1$^{Cre/+}$; vGluT2$^{+/+}$; Rosa26^{tdTom}*) and $V3^{OFF}$ mice. Given the proximity of these neurons, the recordings of the MNs were often lost, but we could still photostimulate them. Also, we often were only able to achieve loose cell attached recordings of the $V3_{VLat}$ IN (n = 3 in $V3^{ON}$ mice and n = 3 in $V3^{OFF}$ mice). Nonetheless, photostimulation of loose-patched $V3_{VLat}$ INs produced direct responses in $V3^{ON}$ (Figure 3Ci) and $V3^{OFF}$ (data not shown) mice. Interestingly, in current clamp, photostimulation of the ipsi-MNs reciprocally elicited responses in the $V3_{VLat}$ INs with amplitudes in these loose patch recordings ranging from 0.5 to 30 mV (mean 11 ± 16 mV; Figure 3Cii; n = 3) and latencies of 3.9 ± 0.8 ms, similar to the latencies seen when stimulating $V3_{VLat}$ INs and recording ipsi-MNs from both $V3^{ON}$ and $V3^{OFF}$ mice. Taken together, these results indicate that there are bidirectional vGlut2-independent connections between $V3_{VLat}$ INs and ipsi-MN with similar latencies from the stimuli.

Although the latencies of the responses were longer than those that would be seen with electrical coupling, and although there were some failures in the responses, the bidirectional vGluT2-independent nature of these connections between $V3_{VLat}$ INs and ipsi-MNs led us to ask whether electrical synapses mediated a component of the bidirectional connection. We therefore recorded $V3_{VLat}$-evoked EPSCs in a subset of MNs from P7–P9 spinal cords before and during application of the gap junction blocker carbenoxolone (CBX) in *Sim1$^{Cre/+}$; Rosa26^{tdTom}* mice. Photostimulation of MNs during CBX application produced direct responses of similar size (range 100–235 pA, mean 143 pA) to those recorded pre-CBX (range 97–233 pA, mean 140 pA), indicating no observable effect of CBX on MN responsiveness ($p > 0.94$). However, during CBX application, there was a significant ~50% reduction in the amplitude of $V3_{VLat}$-evoked EPSCs in ipsi-MNs from 62 pA (range 53–80 pA) to 30 pA (range 25–39 pA) (Figures 3D and 3E; p = 0.008, n = 3). There was no significant change in the latencies of the responses (3.6 ± 0.8 to 4.6 ± 1.0 ms; Figures 3D and 3E; p = 0.053). Although this reduction in amplitude could result from non-specific effects of CBX, we could not rule out a component of electrical synaptic transmission between $V3_{VLat}$ INs and ipsi-MNs at this developmental stage.

We then further explored the MN to $V3_{VLat}$ responses at P13–P14 by stimulating ventral roots in either oblique slices (n = 3 mice) or a dorsal horn removed lumbar spinal cord (n = 1) of $V3^{ON}$ mice. In slices, ventral root stimulation evoked currents in 10 of 21 recorded V3 INs, with latencies of 1.6 to 2.3 ms and amplitudes up to 600 pA (Figures 4A and 4B; range 60–600 pA, mean current 194 ± 180 pA). Analysis of the positions of the recorded cells revealed a striking demarcation in the recorded V3 INs, with only 1 of 8 $V3_{VMed}$ INs responding to VR stimulation (Figure 4B). In contrast, large EPSCs were seen in 9 of 13 $V3_{VLat}$ INs in response to antidromic MN stimulation (Figure 4B), suggesting a relative, within-slice specificity of recurrent excitatory effects to this population of V3 INs.

To determine the degree to which these responses were chemically mediated, we then switched to a calcium-free solution (n = 3) and found that all responses were eliminated. Furthermore, following addition of D-APV (50 μM) and NBQX (3 μM),

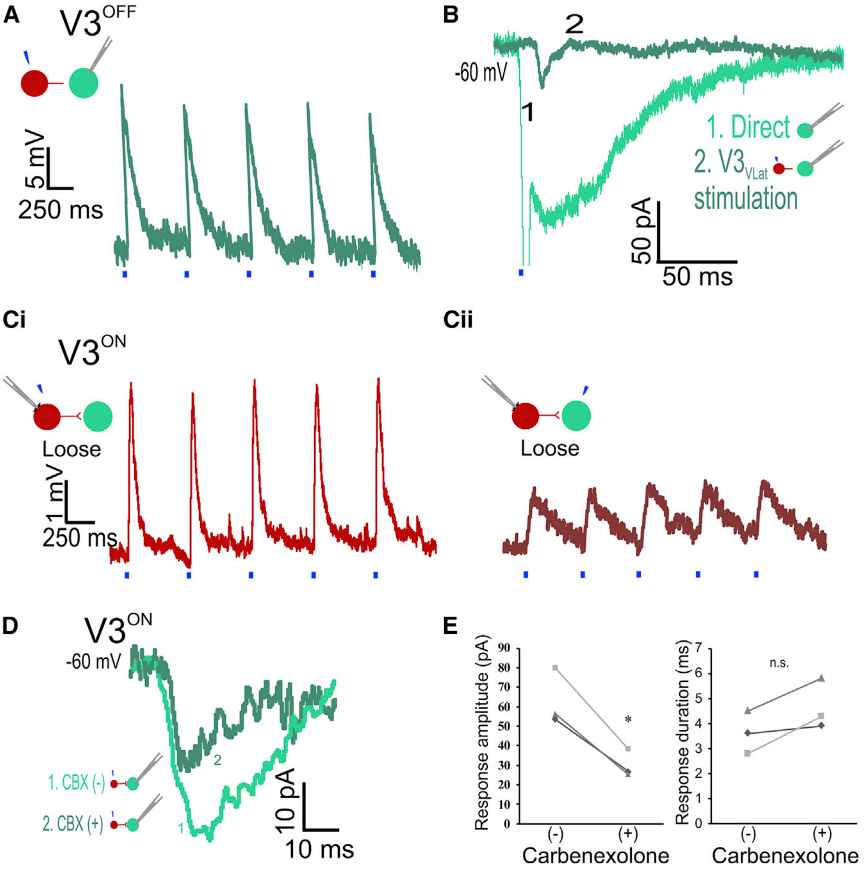

Figure 3. Connectivity between $V3_{VLat}$ INs and Ipsilateral MNs Is Bidirectional

(A) Photostimulation of vGluT2-deleted $V3_{VLat}$ INs elicited EPSPs but not action potentials in ipsilateral MNs.

(B) Voltage-clamp recordings from an MN in response to direct (light green) and vGluT2-deleted $V3_{VLat}$ IN (dark green) photostimulation.

(Ci) Response to direct photostimulation of the loose-patched $V3_{VLat}$ IN ($Sim1^{Cre}$; $Rosa26^{tdTom}$).

(Cii) Photostimulation of the connected MN elicited EPSPs in the loose-patched $V3_{VLat}$ IN.

(D) $V3_{Vlat}$ IN-evoked EPSCs in MNs before (light green) and during CBX (dark green) application.

(E) CBX significantly reduced the response amplitude (n = 3, p = 0.008, pairwise comparison) but not response latency (n = 3, p = 0.053, pairwise comparison).

responses were eliminated in five of six V3 INs (Figure 4C). In the sixth neuron, there was a dramatic reduction in post-synaptic current amplitude without a change in shape, suggesting that there was an incomplete block (data not shown). Although the lack of a clear electrical component could result from one being hidden by the large stimulus artifact, the complete absence of any deflection in all individual traces suggests a lack of electrotonic coupling at this developmental stage. In sum, these data indicate that recurrent excitation of $V3_{VLat}$ INs by motor axon collaterals is mediated largely, if not solely, by glutamatergic transmission.

To study whether these recurrent excitatory effects extended beyond the local circuits in slices, we used a dorsal horn removed preparation, in which stimulation of the L2 ventral root produced monosynaptic responses in 12 of 17 V3 INs distributed in the L2 and caudal L1 segments (Figure S2A). As in the slice experiments, ventral root-evoked EPSCs were recorded in V3 INs located in the more lateral aspect of the spinal cord (n = 8 of 11, mean current 186.4 ± 88.0 pA) but, in addition, were also recorded in 4 of 6 V3 INs located in the more medial spinal cord (mean current 49.3 ± 16.8 pA; Figure S2B). In 6 of the 12 responding V3 INs, we also recorded in current clamp during ventral root stimulation. Ventral root stimulation was capable of eliciting both EPSPs (4 of 6 V3 INs) and action potentials (2 of 6 V3 INs; Figure S2C). Together, these data suggest that recurrent excitation to $V3_{VLat}$ INs extends beyond the 300 μm of a transverse slice, that MN input to V3 INs is sufficient to produce action potentials, and that some recurrent excitation extends to the $V3_{VMed}$ INs as well.

Heteronymous and Homonymous Connectivity of $V3_{VMed}$ INs

We next sought to determine whether $V3_{VLat}$ INs form local connections with other $V3_{VLat}$ or $V3_{VMed}$ INs in *$Sim1^{Cre/+}$; $Rosa26^{tdTom}$* mice. On photostimulating $V3_{VLat}$ INs, we found no responses in recorded V3 INs ($V3_{VMed}$, n = 7; $V3_{VLat}$, n = 5). Thus, $V3_{VLat}$ INs project to ipsi-MNs but not to other local V3 INs.

We next looked for evidence of local connectivity of $V3_{VMed}$ INs to $V3_{VLat}$ INs. We recorded from 13 $V3_{VLat}$ INs and found 16 connections when photostimulating 73 $V3_{VMed}$ INs (Figure 5A). Direct stimulation of the $V3_{VLat}$ INs consistently produced action potentials in these neurons (Figure 5B). Under voltage-clamp configuration, the mean response amplitude of the direct stimulation was 79 ± 39 pA (Figure 5C, light red trace). Conversely, the responses elicited in $V3_{VLat}$ INs from $V3_{VMed}$ IN photostimulation were variable, with both action potentials and EPSPs observed in the same recordings (Figure 5D). In voltage clamp, the response amplitudes ranged from 22 to 140 pA, with a mean amplitude of 84 ± 62 pA (n = 9), similar to that seen with direct photostimulation (Figures 5C and 5E, dark red trace). These connections also demonstrated a degree of convergence with one (n = 9), two (n = 2; Figure 5D), or three (n = 1) individual $V3_{VMed}$ INs eliciting synaptic responses in the recorded $V3_{VLat}$ IN.

To ensure that the $V3_{VMed}$-evoked responses in $V3_{VLat}$ INs were produced by synaptic transmission, we compared latencies between direct activation of the $V3_{VLat}$ INs with those evoked by photostimulation of $V3_{VMed}$ INs. Direct photostimulation of $V3_{VLat}$ INs resulted in responses with a mean latency of 0.2 ± 0.2 ms, whereas the $V3_{VMed}$-evoked responses in $V3_{VLat}$ INs had a significantly longer mean latency of 3.7 ± 1.4 ms (Figures 5C and 5E; p < 0.0001). Rise times were similar (3.4 ± 1.5

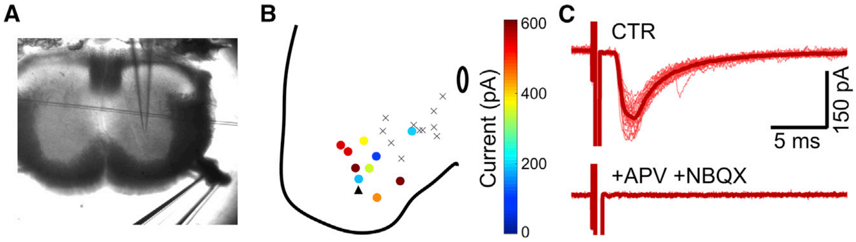

Figure 4. Glutamatergic Recurrent Excitation of V3 INs by Motoneurons

(A) Oblique slice preparation in which a suction electrode was used to stimulate the ventral root while recording from V3 INs.

(B) Composite diagram from seven slices showing location of V3 INs recorded during ventral root stimulation, including those that responded with monosynaptic EPSCs (circles) and those with no responses (X's) to stimulation. Color indicates the size of the EPSCs in the V3 INs in response to ventral root stimulation. Black arrowhead indicates the location of the V3 IN described in (C).

(C) Representative responses recorded in a V3 IN in response to ventral stimulation in control (top trace) and in the presence of glutamatergic antagonists (bottom trace). Stimulus artifacts have been truncated and subtracted (by fitting with a double exponential). Thick red line indicates average response.

See also Figure S2.

versus 4.4 ± 0.4 ms, n = 3, p = 0.13). Of note, the jitter of the onset of each $V3_{VMed}$-evoked EPSC was minimal (variance = 2.3 × 10^{-7} s^2; Figure S3). Together these results indicate that $V3_{VMed}$ INs form synaptic connections with $V3_{VLat}$ INs.

In addition to forming synapses with $V3_{VLat}$ INs, we asked whether $V3_{VMed}$ INs form synaptic connections with other $V3_{VMed}$ INs (Figure 5F). In five recorded $V3_{VMed}$ INs, six connections were established by photostimulating 18 $V3_{VMed}$ INs. Direct photostimulation of the recorded $V3_{VMed}$ IN produced action potentials (Figure 5G). Photostimulation of other $V3_{VMed}$ INs elicited EPSPs with a mean amplitude of 3 ± 4 mV (Figure 5H; n = 6) but never elicited action potentials. Similar to their connectivity with $V3_{VLat}$ INs, there was evidence of convergence, with two of the five post-synaptic $V3_{VMed}$ INs receiving input from two $V3_{VMed}$ INs.

Finally, to determine if these connections are vGluT2 mediated, connectivity between the ventral V3 IN sub-populations was examined in $V3^{OFF}$ mice. No connections between ventral V3 INs were found in $V3^{OFF}$ slices (3 $V3_{VLat}$ INs, 4 $V3_{VMed}$ INs with 40 V3 INs stimulated), suggesting that synaptic connections between ventral V3 INs are purely glutamatergic and that our vGluT2 knockout strategy was likely complete (see above). Taken together, these data indicate that $V3_{VMed}$ INs project to, converge on, and form glutamatergic synapses with both other local $V3_{VMed}$ INs and local $V3_{VLat}$ INs, but $V3_{VLat}$ INs do not project to other local V3 INs.

Ventral V3 INs Form Layered Microcircuits with Medial-to-Lateral Processing

On the basis of the connectivity patterns described above, we next sought to determine whether, within a single slice, there is local layered connectivity from $V3_{VMed}$ INs to $V3_{VLat}$ INs, which in turn project to ipsi-MNs (Figure 6A). In a subset of experiments (n = 3), action potentials in MNs were initially recorded in response to direct photostimulation (Figure 6B). We then selected a $V3_{VLat}$ IN that, when photostimulated, produced action potentials in the recorded MN (Figure 6C). We proceeded with loose patch-clamp recordings of these $V3_{VLat}$ INs and photostimulated $V3_{VMed}$ INs. In all experiments, photostimulation of a $V3_{VMed}$ IN elicited EPSPs in the recorded $V3_{VLat}$ IN that produced synaptic responses in the MN (Figure 6D). This demonstrates local, layered connectivity in which synaptic processing proceeds from medial to lateral in the ventral spinal cord.

DISCUSSION

Motor circuits rely on spinal interneuronal processing to coordinate and regulate motor output. However, the organizational logic of these circuits (i.e., the basic wiring diagram at a cellular level) remains unknown. To address this, we used a spatial light modulator (SLM) system to activate individual genetically defined neurons while recording from putative targets. In doing so, we identified a local circuit composed of layered glutamatergic connectivity from spinal $V3_{VMed}$ to $V3_{VLat}$ INs, which in turn form synapses with ipsi-MNs. The MNs, in turn, project back to V3 INs, forming recurrent, glutamatergic connections (Figure 7). As most of our experiments were carried out in acute spinal cord slices, we cannot exclude that V3 INs form synaptic contacts beyond the slices. Nevertheless, it is clear that ventral V3 INs form focal, layered microcircuits. Although this functional layered structure is analogous in principle to that of cortical columns, we refer to these local circuits as "modules," as the word "column" has other connotations in the spinal cord (e.g., "spinal column" or "motor column"). These V3 IN modules are layered microcircuits that are substrates for medial to lateral synaptic processing in motor circuits.

Diversity of Ventral V3 INs

Although previous studies have resolved dorsal and ventral sub-populations of V3 INs on the basis of electrophysiological properties, location, and morphology (Borowska et al., 2013, 2015), it was not clear until we began to examine connectivity that the ventral population can further be subdivided into at least two additional sub-populations. Diversity of cardinal classes of spinal INs is becoming increasingly clear. For example, a small fraction of V0 INs, the cholinergic $V0_C$ sub-population, acts to control the input:output gain of MNs (Miles et al., 2007; Zagoraiou et al., 2009), whereas $V0_D$ and $V0_V$ INs have roles in left-right coordination (Talpalar et al., 2013). In addition, V1 INs can be sub-classified into approximately 50 populations on the basis of their combinatorial expression of various transcription factors, electrophysiological properties, and location (Bikoff et al., 2016). And V2a INs can similarly be subdivided into multiple classes (Hayashi et al., 2018). Therefore, data from experiments in which whole classes are manipulated (e.g., Crone et al., 2008; Gosgnach et al., 2006; Lanuza et al., 2004; Zhang et al., 2014, 2008) should be interpreted with the knowledge that many, possibly

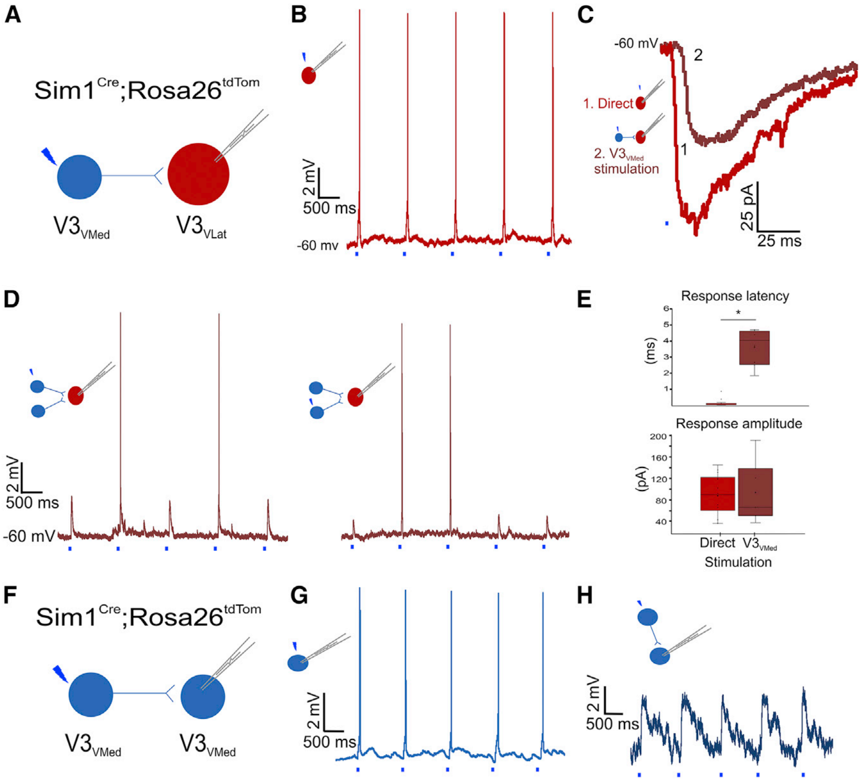

Figure 5. $V3_{VMed}$ INs Form Synapses with $V3_{VLat}$ INs and Other $V3_{VMed}$ INs

(A) $V3_{VMed}$ INs were photostimulated while recording $V3_{VLat}$ INs (n = 13).

(B) Direct photostimulation of the patched $V3_{VLat}$ INs elicited a single action potential per stimulation.

(C) Voltage-clamp recordings from a $V3_{VLat}$ IN in response to direct (light red) and $V3_{VMed}$ IN (dark red) photostimulation.

(D) Photostimulation of two different $V3_{VMed}$ INs produced EPSPs or action potentials in the same recorded $V3_{VLat}$ IN.

(E) Increased response latency with $V3_{VMed}$ IN photostimulation (dark red) compared to direct $V3_{VLat}$ IN photostimulation (light red; n = 9, $p < 0.0001$, Student's t test), but no difference in response amplitude (n = 9, p = 0.4, Student's t test). Data shown are mean and quartile range, with circles indicating individual responses.

(F) $V3_{VMed}$ INs were photostimulated while recording other $V3_{VMed}$ INs.

(G) Direct photostimulation of the patched $V3_{VMed}$ elicited action potentials.

(H) Photostimulation of $V3_{VMed}$ INs produced EPSPs in the recorded $V3_{VMed}$ INs.

See also Figure S5.

diverse, populations of INs are being affected. To date, molecular markers for sub-types of V3 INs have not been identified, and molecularly distinguishing different types of ventral V3 INs has been particularly problematic (unpublished data). Here, we use location, size, and connectivity patterns to further classify the ventral V3 IN population into $V3_{VMed}$ and $V3_{VLat}$ INs and to understand connectivity of these sub-classes.

Ipsilateral Connectivity of Commissural V3 INs

V3 INs have been defined as a cardinal class of excitatory INs with primarily commissural projections (Blacklaws et al., 2015; Zhang et al., 2008). We were thus somewhat surprised to find ipsilateral connectivity between $V3_{VMed}$ INs, $V3_{VLat}$ INs, and MNs, and then to establish that these INs are not a separate population of non-commissural V3 INs, but rather that these INs project bilaterally. Although we have not identified the contralateral targets of V3 INs, we found no connections with contralateral MNs within the slice, possibly because the axons ascend or descend after passing through the ventral commissure (Blacklaws et al., 2015; Zhang et al., 2008). It is thus likely that these axons would have been truncated in our slices, leading to the inability to identify their contralateral targets, whether MNs or INs, in this preparation.

Bilateral projections of V3 INs could serve to coordinate right-left synergistic activity across different segments during locomotion, as suggested by previous physiological (Zhang et al., 2008) and modeling studies (Danner et al., 2017; Shevtsova et al., 2015). Such bilateral synergistic excitation is consistent with the proposition that a V3 excitatory commissural pathway serves to balance locomotor output across the spinal cord, in contrast to inhibitory commissural pathways that coordinate the phasing of left and right locomotor circuits (Zhang et al., 2008; Danner et al., 2017). However, in the absence of genetic access to particular sub-populations of V3 INs, such as those studied here, the roles of these specific IN populations cannot be determined.

MN-Mediated Recurrent Excitation of V3 INs

We have shown here that MNs monosynaptically excite ventral V3 INs via glutamatergic neurotransmission. Although it has been established that MNs have recurrent axon collaterals that form synapses with Renshaw cells (Eccles et al., 1954) and other MNs (Bhumbra and Beato, 2018), other targets of MNs have not been demonstrated. But it has been shown that in the mouse, motor axon stimulation can initiate locomotor-like activity (Mentis et al., 2005), and optogenetic activation or inactivation of MNs influences locomotor rhythm through a glutamatergic pathway, presumably constituting a class of unidentified excitatory INs (Falgairolle et al., 2017). Because V3 INs contribute to the robustness of the locomotor rhythm (Zhang et al., 2008) and influence the rhythmogenic component of locomotor circuits (Shevtsova et al., 2015), we hypothesize that the influence of MNs on the locomotor rhythm is at least partly mediated by the recurrent excitation of V3 INs by MNs as described here. Taking into account the bilateral projections of V3 INs, this "retrograde" connectivity could function as a feedforward mechanism by which MNs provide excitatory drive to both the contralateral and ipsilateral spinal cord.

But this feedforward excitatory microcircuit defines a positive feedback loop, and positive feedback typically leads to

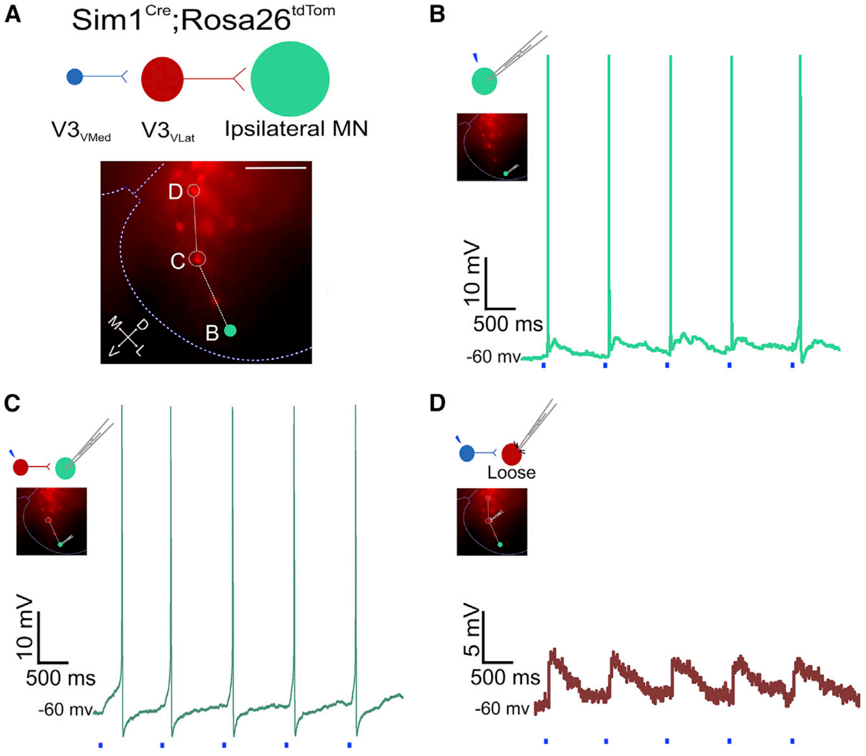

Figure 6. Local Spinal Layers between V3 INs and MNs

(A) Systematic investigation of $V3_{VMed}$ → $V3_{VLat}$ → MN connectivity. Image obtained during patch-clamp recordings. Letters B–D depict the order in which neurons were patched and stimulated to demonstrate the layered connectivity within a slice (n = 3). M, medial; L, lateral; D, dorsal; V, ventral.

(B) Direct photostimulation of the patched MN elicited one action potential per stimulus.

(C) Photostimulating a $V3_{VLat}$ IN elicited a single action potential per stimulus in the recorded MN.

(D) Photostimulating a $V3_{VMed}$ IN elicited EPSPs in the loose-patched $V3_{VLat}$ IN that was stimulated in (C).

Scale bars, 100 μm.

oscillatory activity and instability. There are other examples of positive feedback loops in motor systems, including MN to MN connectivity (Bhumbra and Beato, 2018) and group Ib afferent excitatory pathways mediating force feedback (Prochazka et al., 1997a). This latter pathway may, counterintuitively, stabilize movement (Prochazka et al., 1997b). It will be interesting to determine whether this V3 IN positive feedback loop contributes to motor stability (cf. Zhang et al., 2008) and/or whether this loop leads to oscillatory activity in MNs during rhythmic behaviors. Interestingly, this positive feedback loop is in parallel with a negative feedback loop composed of Renshaw cells. Given the different inputs and cell-specific intrinsic and integrative properties of these different neuron types, it would be unlikely that these two pathways would simply cancel each other out; the nuances of the function of these two parallel loops and their contributions to motor output during behavior remain unknown and will require detailed knowledge of their activity and connectivity patterns.

Interestingly, transmission from MNs to V3 INs is purely glutamatergic. Although activation of muscle fibers by MNs is mediated by acetylcholine, MN activation of Renshaw cells is via mixed cholinergic-glutamatergic transmission (Nishimaru et al., 2005, Mentis et al., 2005, Lamotte-d'Incamps et al., 2017). In addition, recurrent excitation between MNs is purely glutamatergic (Bhumbra and Beato, 2018). Thus, recurrent excitation of V3 INs is similar to that of MNs. Whether the segregation of transmitter systems as a function of the post-synaptic targets is due to pre- and/or post-synaptic mechanisms is not yet clear, but at least for motor axon synapses with Renshaw cells, it appears that the two transmitter systems are segregated at the level of individual contacts, even though unitary EPSCs are invariably mixed (Lamotte d'Incamps et al., 2017).

Local Ventral V3 IN Modules and Layered Spinal Processing

Modular layered circuits are of course not unique to the spinal cord. Perhaps the prototypical example of a layered structure is the mammalian cerebral cortex, in which molecularly defined populations of neurons are beautifully distributed into layers (Narayanan et al., 2017). This layering is thought to form the basis for the formation of canonical microcircuits (Douglas and Martin, 2004). However, organized functional processing can occur in the absence of structural layers such as in the bird pallium (Calabrese and Woolley, 2015) or in reeler mutant mice in which structural layers are disrupted (Guy and Staiger, 2017). These studies highlight the importance of understanding circuits from a functional connectivity point of view rather than from a pure structural, laminar point of view and may provide insight into the function of layered V3 IN modules.

Many last-order INs (INs that project directly to MNs) have been identified over the past decades using electrophysiological techniques in the cat and genetic techniques in the mouse (see Brownstone and Bui, 2010, for a review), but few oligosynaptic spinal pathways have been defined. Genetic techniques in the mouse have provided knowledge about connectivity of cardinal classes of INs with MNs, using either anatomical (e.g., Al-Mosawie et al., 2007; Dougherty and Kiehn, 2010; Zhang et al., 2008) or monosynaptic rabies virus tracing (e.g., Stepien et al., 2010) techniques. In addition, a few examples of local connectivity between identified INs have been reported (Dougherty et al., 2013; Hinckley and Ziskind-Conhaim, 2006; Wilson et al., 2007; Zhong et al., 2010). However, perhaps with the exception of Renshaw cells that project to Ia inhibitory INs that in turn project to MNs (Alvarez and Fyffe, 2007), layered multi-synaptic processing involving discrete IN populations has not been directly demonstrated. Nonetheless, experimental evidence indicates that oligosynaptic processing in the spinal cord does occur (e.g., flexor reflex afferent circuits; Andén et al., 1964, 1966; Jankowska

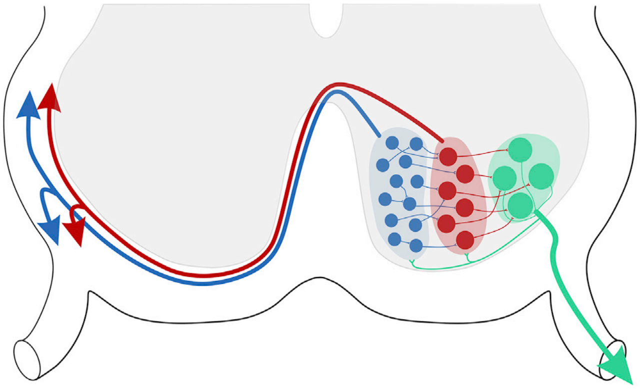

Figure 7. Local Layered V3 IN Microcircuits Involving $V3_{VMed}$ INs (Blue), $V3_{VLat}$ INs (Red), and MNs (Green)

Synaptic processing occurs in medial to lateral modules with glutamatergic synapses between adjacent $V3_{VMed}$ INs, from $V3_{VMed}$ INs to $V3_{VLat}$ INs, from $V3_{VLat}$ INs to ipsilateral MNs, and glutamatergic recurrent excitation of V3 INs by ipsilateral MNs.

et al., 1967), a finding supported by computational modeling (Danner et al., 2017). Here, we have combined genetic identification of neurons with photostimulation techniques to map a multisynaptic spinal microcircuit (Figure 7).

We suggest that the convergent layered V3 IN modules described here function similarly to the prototypical example of layered structures in the primary somatosensory (barrel) cortex, in which discrete sensory information from facial whiskers maps onto the cortex. These layers have specific intralaminar (akin to $V3_{VMed}$ IN interconnectivity), translaminar (akin to $V3_{VMed}$-to-$V3_{VLat}$ IN connectivity), and transcolumnar (akin to crossed projections of ventral V3 INs) connectivity (Schubert et al., 2007). It has been proposed that the layered modules in barrel cortex separate a spatially specific, segregated organization for sensory input from an integrating, coincidence detection function with low spatial specificity; together, these layers would provide the substrate for spatiotemporal sensory function (Schubert et al., 2007).

Our present work indicates that there could be a similar arrangement in ventral V3 IN modules—but reversed in order for this motor system—in which $V3_{VMed}$ INs function to integrate central motor commands, and $V3_{VLat}$ INs segregate the output to specific motor pools. In support of this hypothesis, the $V3_{VMed}$ IN population is located in the ventromedial spinal cord, an area that receives direct descending motor commands (e.g., reticulospinal) that initiate movement (Matsuyama et al., 1988). Reticulospinal innervation of the spinal cord is broad and extensively branched in multiple spinal cord segments (Peterson et al., 1975) and may thus have relatively low spatial (motor pool) specificity. Indeed, the specificity of the reticulospinal system in terms of muscle group activation is less than that of both the motor cortex and the spinal cord in non-human primates (Zaaimi et al., 2018). On the other hand, last-order INs tend to innervate more restricted motor pools, requiring coordination of multiple premotor INs to activate specific muscle synergies (Takei et al., 2017). Thus, the reticulospinal system may be responsible for "priming" $V3_{VMed}$ INs, which could serve as local integrating neurons that project to spatially specific, segregated $V3_{VLat}$ INs that provide motor specificity (cf. Zaaimi et al., 2018).

In the absence of genetic access to the different sub-populations, we cannot specifically define the function of these two populations of ventral V3 INs. However, the organization described here and the hypothesis presented above are reminiscent of proposed two-layered spinal circuits for locomotion, in which the first layer is involved in the generation of the locomotor rhythm and projects to a second layer responsible for pattern formation (Brownstone and Wilson, 2008; Rybak et al., 2006). If ventral V3 INs are organized into rhythm- and pattern-generating layers, then we would predict that removal of the $V3_{VMed}$ IN population would replicate the instability of the locomotor rhythm seen following elimination of all V3 INs (Zhang et al., 2008), whereas removing $V3_{VLat}$ INs could lead to a reduction in power and specificity of motor pool activation.

In summary, using a combination of genetic strategies, photostimulation, and electrophysiology, we studied cell-to-cell connectivity and have revealed a local pathway that is capable of processing information in functional layers of intra- and interconnected modules of V3 INs and MNs in the spinal cord. Such a system is in line with layered computational frameworks in the cerebral cortex and other CNS regions, which provide a common principle of CNS organization that ensures accuracy and flexibility of information processing.

STAR★METHODS

Detailed methods are provided in the online version of this paper and include the following:

- KEY RESOURCES TABLE
- CONTACT FOR REAGENT AND RESOURCE SHARING
- EXPERIMENTAL MODEL AND SUBJECT DETAILS
  - Mice
- METHOD DETAILS
  - Slice preparation
  - Whole-cell patch-clamp recordings and stimulation
  - MNI-glutamate perfusion and photostimulation protocol
  - Dextran labeling
  - Carbenoxolone (CBX) perfusion
  - Immunofluorescence and imaging
- QUANTIFICATION AND STATISTICAL ANALYSIS
- DATA AND SOFTWARE AVAILABILITY

SUPPLEMENTAL INFORMATION

Supplemental Information includes three figures and can be found with this article online at https://doi.org/10.1016/j.celrep.2018.08.095.

ACKNOWLEDGMENTS

We thank Dallas Bennett, Dylan Deska-Gauthier, and Nadine Simons-Weidenmaier for animal care and technical support and Dr. Amanda Pocratsky for helpful comments and feedback. The research was funded by the Canadian Health Institutes of Research (MOP-110950 to Y.Z. and MOP-136981 to R.M.B. and Y.Z.), Wellcome (110193 to R.M.B.), and the Medical Research Council (MR/R011494 to M.B. and R.M.B.). Equipment used for

photostimulation experiments was funded by the Canadian Foundation for Innovation (228190 to R.M.B.) and the Nova Scotia Research and Innovation Trust (R.M.B.). R.M.B. is supported by Brain Research UK.

AUTHOR CONTRIBUTIONS

Methodology, J.W.C., F.N., M.B., R.M.B., and Y.Z.; Formal Analysis, J.W.C.; Validation, J.W.C.; Investigation, J.W.C., F.N.; Data Curation, J.W.C., M.B.; Writing – Original Draft, J.W.C., R.M.B.; Writing – Review & Editing, J.W.C., M.B., R.M.B., Y.Z.; Data Curation & Analysis, M.B.; Funding Acquisition, R.M.B., Y.Z.; Supervision, R.M.B., Y.Z.; Conceptualization, R.M.B., Y.Z.; Resources, R.M.B., Y.Z.

DECLARATION OF INTERESTS

The authors declare no competing interests.

Received: April 17, 2018
Revised: July 25, 2018
Accepted: August 30, 2018
Published: October 2, 2018

REFERENCES

Al-Mosawie, A., Wilson, J.M., and Brownstone, R.M. (2007). Heterogeneity of V2-derived interneurons in the adult mouse spinal cord. Eur. J. Neurosci. *26*, 3003–3015.

Alvarez, F.J., and Fyffe, R.E.W. (2007). The continuing case for the Renshaw cell. J. Physiol. *584*, 31–45.

Andén, N.E., Jukes, M.G.M., Lundberg, A., and Vyklický, L. (1964). A new spinal flexor reflex. Nature *202*, 1344–1345.

Andén, N.-E., Jukes, M.G.M., Lundberg, A., and Vyklický, L. (1966). The effect of DOPA on the spinal cord. 1. Influence on transmission from primary afferents. Acta Physiol. Scand. *67*, 373–386.

Andersson, L.S., Larhammar, M., Memic, F., Wootz, H., Schwochow, D., Rubin, C.J., Patra, K., Arnason, T., Wellbring, L., Hjälm, G., et al. (2012). Mutations in DMRT3 affect locomotion in horses and spinal circuit function in mice. Nature *488*, 642–646.

Bhumbra, G.S., and Beato, M. (2018). Recurrent excitation between motoneurones propagates across segments and is purely glutamatergic. PLoS Biol. *16*, e2003586.

Bhumbra, G.S., Bannatyne, B.A., Watanabe, M., Todd, A.J., Maxwell, D.J., and Beato, M. (2014). The recurrent case for the Renshaw cell. J. Neurosci. *34*, 12919–12932.

Bikoff, J.B., Gabitto, M.I., Rivard, A.F., Drobac, E., Machado, T.A., Miri, A., Brenner-Morton, S., Famojure, E., Diaz, C., Alvarez, F.J., et al. (2016). Spinal inhibitory interneuron diversity delineates variant motor microcircuits. Cell *165*, 207–219.

Birinyi, A., Viszokay, K., Wéber, I., Kiehn, O., and Antal, M. (2003). Synaptic targets of commissural interneurons in the lumbar spinal cord of neonatal rats. J. Comp. Neurol. *461*, 429–440.

Blacklaws, J., Deska-Gauthier, D., Jones, C.T., Petracca, Y.L., Liu, M., Zhang, H., Fawcett, J.P., Glover, J.C., Lanuza, G.M., and Zhang, Y. (2015). Sim1 is required for the migration and axonal projections of V3 interneurons in the developing mouse spinal cord. Dev. Neurobiol. *75*, 1003–1017.

Borowska, J., Jones, C.T., Zhang, H., Blacklaws, J., Goulding, M., and Zhang, Y. (2013). Functional subpopulations of V3 interneurons in the mature mouse spinal cord. J. Neurosci. *33*, 18553–18565.

Borowska, J., Jones, C.T., Deska-Gauthier, D., and Zhang, Y. (2015). V3 interneuron subpopulations in the mouse spinal cord undergo distinctive postnatal maturation processes. Neuroscience *295*, 221–228.

Brownstone, R.M., and Bui, T.V. (2010). Spinal interneurons providing input to the final common path during locomotion. Prog. Brain Res. *187*, 81–95.

Brownstone, R.M., and Wilson, J.M. (2008). Strategies for delineating spinal locomotor rhythm-generating networks and the possible role of Hb9 interneurones in rhythmogenesis. Brain Res. Brain Res. Rev. *57*, 64–76.

Bui, T.V., Akay, T., Loubani, O., Hnasko, T.S., Jessell, T.M., and Brownstone, R.M. (2013). Circuits for grasping: spinal dI3 interneurons mediate cutaneous control of motor behavior. Neuron *78*, 191–204.

Calabrese, A., and Woolley, S.M.N. (2015). Coding principles of the canonical cortical microcircuit in the avian brain. Proc. Natl. Acad. Sci. U S A *112*, 3517–3522.

Caldeira, V., Dougherty, K.J., Borgius, L., and Kiehn, O. (2017). Spinal Hb9:Cre-derived excitatory interneurons contribute to rhythm generation in the mouse. Sci. Rep. *7*, 41369.

Crone, S.A., Quinlan, K.A., Zagoraiou, L., Droho, S., Restrepo, C.E., Lundfald, L., Endo, T., Setlak, J., Jessell, T.M., Kiehn, O., and Sharma, K. (2008). Genetic ablation of V2a ipsilateral interneurons disrupts left-right locomotor coordination in mammalian spinal cord. Neuron *60*, 70–83.

Crone, S.A., Zhong, G., Harris-Warrick, R., and Sharma, K. (2009). In mice lacking V2a interneurons, gait depends on speed of locomotion. J. Neurosci. *29*, 7098–7109.

Danner, S.M., Shevtsova, N.A., Frigon, A., and Rybak, I.A. (2017). Computational modeling of spinal circuits controlling limb coordination and gaits in quadrupeds. eLife *6*, 6.

Dougherty, K.J., and Kiehn, O. (2010). Firing and cellular properties of V2a interneurons in the rodent spinal cord. J. Neurosci. *30*, 24–37.

Dougherty, K.J., Zagoraiou, L., Satoh, D., Rozani, I., Doobar, S., Arber, S., Jessell, T.M., and Kiehn, O. (2013). Locomotor rhythm generation linked to the output of spinal shox2 excitatory interneurons. Neuron *80*, 920–933.

Douglas, R.J., and Martin, K.A.C. (2004). Neuronal circuits of the neocortex. Annu. Rev. Neurosci. *27*, 419–451.

Dyck, J., Lanuza, G.M., and Gosgnach, S. (2012). Functional characterization of dI6 interneurons in the neonatal mouse spinal cord. J. Neurophysiol. *107*, 3256–3266.

Eccles, J.C., Fatt, P., and Koketsu, K. (1954). Cholinergic and inhibitory synapses in a pathway from motor-axon collaterals to motoneurones. J. Physiol. *126*, 524–562.

Enjin, A., Perry, S., Hilscher, M.M., Nagaraja, C., Larhammar, M., Gezelius, H., Eriksson, A., Leão, K.E., and Kullander, K. (2017). Developmental disruption of recurrent inhibitory feedback results in compensatory adaptation in the Renshaw cell-motor neuron circuit. J. Neurosci. *37*, 5634–5647.

Falgairolle, M., Puhl, J.G., Pujala, A., Liu, W., and O'Donovan, M.J. (2017). Motoneurons regulate the central pattern generator during drug-induced locomotor-like activity in the neonatal mouse. eLife *6*, 6.

Gosgnach, S., Lanuza, G.M., Butt, S.J.B., Saueressig, H., Zhang, Y., Velasquez, T., Riethmacher, D., Callaway, E.M., Kiehn, O., and Goulding, M. (2006). V1 spinal neurons regulate the speed of vertebrate locomotor outputs. Nature *440*, 215–219.

Griener, A., Zhang, W., Kao, H., Haque, F., and Gosgnach, S. (2017). Anatomical and electrophysiological characterization of a population of dI6 interneurons in the neonatal mouse spinal cord. Neuroscience *362*, 47–59.

Guy, J., and Staiger, J.F. (2017). The functioning of a cortex without layers. Front. Neuroanat. *11*, 54.

Hayashi, M., Hinckley, C.A., Driscoll, S.P., Moore, N.J., Levine, A.J., Hilde, K.L., Sharma, K., and Pfaff, S.L. (2018). Graded arrays of spinal and supraspinal V2a interneuron subtypes underlie forelimb and hindlimb motor control. Neuron *97*, 869–884.

Hinckley, C.A., and Ziskind-Conhaim, L. (2006). Electrical coupling between locomotor-related excitatory interneurons in the mammalian spinal cord. J. Neurosci. *26*, 8477–8483.

Hnasko, T.S., Chuhma, N., Zhang, H., Goh, G.Y., Sulzer, D., Palmiter, R.D., Rayport, S., and Edwards, R.H. (2010). Vesicular glutamate transport promotes dopamine storage and glutamate corelease in vivo. Neuron *65*, 643–656.

Jankowska, E., Jukes, M.G.M., Lund, S., and Lundberg, A. (1967). The effect of DOPA on the spinal cord. 5. Reciprocal organization of pathways transmitting excitatory action to alpha motoneurones of flexors and extensors. Acta Physiol. Scand. *70*, 369–388.

Jessell, T.M. (2000). Neuronal specification in the spinal cord: inductive signals and transcriptional codes. Nat. Rev. Genet. *1*, 20–29.

Kiehn, O. (2016). Decoding the organization of spinal circuits that control locomotion. Nat. Rev. Neurosci. *17*, 224–238.

Lafreniere-Roula, M., and McCrea, D.A. (2005). Deletions of rhythmic motoneuron activity during fictive locomotion and scratch provide clues to the organization of the mammalian central pattern generator. J. Neurophysiol. *94*, 1120–1132.

Lamotte d'Incamps, B., Bhumbra, G.S., Foster, J.D., Beato, M., and Ascher, P. (2017). Segregation of glutamatergic and cholinergic transmission at the mixed motoneuron Renshaw cell synapse. Sci. Rep. *7*, 4037.

Lanuza, G.M., Gosgnach, S., Pierani, A., Jessell, T.M., and Goulding, M. (2004). Genetic identification of spinal interneurons that coordinate left-right locomotor activity necessary for walking movements. Neuron *42*, 375–386.

Lutz, C., Otis, T.S., DeSars, V., Charpak, S., DiGregorio, D.A., and Emiliani, V. (2008). Holographic photolysis of caged neurotransmitters. Nat. Methods *5*, 821–827.

Matsuyama, K., Ohta, Y., and Mori, S. (1988). Ascending and descending projections of the nucleus reticularis gigantocellularis in the cat demonstrated by the anterograde neural tracer, Phaseolus vulgaris leucoagglutinin (PHA-L). Brain Res. *460*, 124–141.

Mentis, G.Z., Alvarez, F.J., Bonnot, A., Richards, D.S., Gonzalez-Forero, D., Zerda, R., and O'Donovan, M.J. (2005). Noncholinergic excitatory actions of motoneurons in the neonatal mammalian spinal cord. Proc. Natl. Acad. Sci. U S A *102*, 7344–7349.

Miles, G.B., Hartley, R., Todd, A.J., and Brownstone, R.M. (2007). Spinal cholinergic interneurons regulate the excitability of motoneurons during locomotion. Proc. Natl. Acad. Sci. U S A *104*, 2448–2453.

Narayanan, R.T., Udvary, D., and Oberlaender, M. (2017). Cell type-specific structural organization of the six layers in rat barrel cortex. Front. Neuroanat. *11*, 91.

Nishimaru, H., Restrepo, C.E., Ryge, J., Yanagawa, Y., and Kiehn, O. (2005). Mammalian motor neurons corelease glutamate and acetylcholine at central synapses. Proc. Natl. Acad. Sci. U S A *102*, 5245–5249.

Peterson, B.W., Maunz, R.A., Pitts, N.G., and Mackel, R.G. (1975). Patterns of projection and braching of reticulospinal neurons. Exp. Brain Res. *23*, 333–351.

Prochazka, A., Gillard, D., and Bennett, D.J. (1997a). Positive force feedback control of muscles. J. Neurophysiol. *77*, 3226–3236.

Prochazka, A., Gillard, D., and Bennett, D.J. (1997b). Implications of positive feedback in the control of movement. J. Neurophysiol. *77*, 3237–3251.

Rybak, I.A., Stecina, K., Shevtsova, N.A., and McCrea, D.A. (2006). Modelling spinal circuitry involved in locomotor pattern generation: insights from the effects of afferent stimulation. J. Physiol. *577*, 641–658.

Schubert, D., Kötter, R., and Staiger, J.F. (2007). Mapping functional connectivity in barrel-related columns reveals layer- and cell type-specific microcircuits. Brain Struct. Funct. *212*, 107–119.

Shevtsova, N.A., Talpalar, A.E., Markin, S.N., Harris-Warrick, R.M., Kiehn, O., and Rybak, I.A. (2015). Organization of left-right coordination of neuronal activity in the mammalian spinal cord: Insights from computational modelling. J. Physiol. *593*, 2403–2426.

Shipp, S. (2007). Structure and function of the cerebral cortex. Curr. Biol. *17*, R443–R449.

Stepien, A.E., Tripodi, M., and Arber, S. (2010). Monosynaptic rabies virus reveals premotor network organization and synaptic specificity of cholinergic partition cells. Neuron *68*, 456–472.

Takei, T., Confais, J., Tomatsu, S., Oya, T., and Seki, K. (2017). Neural basis for hand muscle synergies in the primate spinal cord. Proc. Natl. Acad. Sci. U S A *114*, 8643–8648.

Talpalar, A.E., Bouvier, J., Borgius, L., Fortin, G., Pierani, A., and Kiehn, O. (2013). Dual-mode operation of neuronal networks involved in left-right alternation. Nature *500*, 85–88.

Wilson, J.M., Cowan, A.I., and Brownstone, R.M. (2007). Heterogeneous electrotonic coupling and synchronization of rhythmic bursting activity in mouse Hb9 interneurons. J. Neurophysiol. *98*, 2370–2381.

Wilson, J.M., Hartley, R., Maxwell, D.J., Todd, A.J., Lieberam, I., Kaltschmidt, J.A., Yoshida, Y., Jessell, T.M., and Brownstone, R.M. (2005). Conditional rhythmicity of ventral spinal interneurons defined by expression of the Hb9 homeodomain protein. J. Neurosci. *25*, 5710–5719.

Zaaimi, B., Dean, L.R., and Baker, S.N. (2018). Different contributions of primary motor cortex, reticular formation, and spinal cord to fractionated muscle activation. J. Neurophysiol. *119*, 235–250.

Zagoraiou, L., Akay, T., Martin, J.F., Brownstone, R.M., Jessell, T.M., and Miles, G.B. (2009). A cluster of cholinergic premotor interneurons modulates mouse locomotor activity. Neuron *64*, 645–662.

Zahid, M., Vélez-Fort, M., Papagiakoumou, E., Ventalon, C., Angulo, M.C., and Emiliani, V. (2010). Holographic photolysis for multiple cell stimulation in mouse hippocampal slices. PLoS ONE *5*, e9431.

Zhang, Y., Narayan, S., Geiman, E., Lanuza, G.M., Velasquez, T., Shanks, B., Akay, T., Dyck, J., Pearson, K., Gosgnach, S., et al. (2008). V3 spinal neurons establish a robust and balanced locomotor rhythm during walking. Neuron *60*, 84–96.

Zhang, J., Lanuza, G.M., Britz, O., Wang, Z., Siembab, V.C., Zhang, Y., Velasquez, T., Alvarez, F.J., Frank, E., and Goulding, M. (2014). V1 and v2b interneurons secure the alternating flexor-extensor motor activity mice require for limbed locomotion. Neuron *82*, 138–150.

Zhong, G., Droho, S., Crone, S.A., Dietz, S., Kwan, A.C., Webb, W.W., Sharma, K., and Harris-Warrick, R.M. (2010). Electrophysiological characterization of V2a interneurons and their locomotor-related activity in the neonatal mouse spinal cord. J. Neurosci. *30*, 170–182.

Zhong, G., Shevtsova, N.A., Rybak, I.A., and Harris-Warrick, R.M. (2012). Neuronal activity in the isolated mouse spinal cord during spontaneous deletions in fictive locomotion: insights into locomotor central pattern generator organization. J. Physiol. *590*, 4735–4759.

STAR★METHODS

KEY RESOURCES TABLE

REAGENT or RESOURCE	SOURCE	IDENTIFIER
Antibodies		
Rabbit polyclonal anti-DsRed	Clontech	Cat#632496<br>RRID: AB_10013483
Mouse polyclonal anti-Choline Acetytransferase	Millipore	Cat# 632496<br>RRID: AB_2079751
Chemicals, Peptides, and Recombinant Proteins		
Dextran amine (10,000MW)	Thermo Fisher Scientific	Cat# D1956
MNI-caged-L-glutamate	Tocris	Cat#1490
Lucifer yellow dilithium salt	Thermo Fisher Scientifc	Cat# A-5751,<br>RRID: AB_2536191
Neurobiotin	Vector Laboratories	Cat# SP-1120-20<br>RRID: AB_2536191
Carbenoxolone	Sigma-Aldrich	Cat# C4790
NBQX disodium salt hydrate	Sigma-Aldrich	Cat# N185
D-APV	Sigma-Aldrich	Cat# A8054
Experimental Models: Organisms/Strains		
Sim1-Cre	Zhang et al., 2008	N/A
HB9::eGFP	Wilson et al., 2005	N/A
(Ai14) B6.Cg-Gt(ROSA)26Sor$^{tm14(CAG0tdTomotato)Hze}$	The Jackson Laboratory	Jax# 007914<br>MGI: 3809524
vGluT2$^{flox/flox}$ (Slc17a6)	Hnasko et al., 2010<br>Bui et al., 2013	N/A
Software and Algorithms		
Slidebook	Intelligent Imaging Innovation	RRID: SCR_014300
Signal software	Cambridge electronic devices	http://ced.co.uk/us/products/sigovin
Zen	Zeiss	RRID: SCR_013672
Sigma Plot v14.0	Systat software	RRID: SCR_003210

CONTACT FOR REAGENT AND RESOURCE SHARING

Further information and requests for resources and reagents should be directed to and will be fulfilled by the Lead Contact, Robert Browstone (r.brownstone@ucl.ac.uk).

EXPERIMENTAL MODEL AND SUBJECT DETAILS

Mice

Female and male mice (P7-P14) from the following mice lines were used for experimentation: 1) *Sim1*$^{Cre/+;}$ *Rosa26*$^{floxstopTdTom/+}$ (Zhang et al., 2008) to visualize V3 INs, 2) *Sim1*$^{Cre/+}$*;Rosa26*$^{floxstopTdTom/+}$*:HB9::eGFP* mice to visualize both V3 INs and MNs, and 3) *Sim1*$^{Cre/+}$*;vGluT2*$^{flox/flox}$ in which vGluT2 (Slc17a6) is conditionally knocked-out (referred to as V3^{OFF}, Bui et al., 2013; Hnasko et al., 2010).

Animal procedures done at Dalhousie University were approved by the University Committee on Laboratory Animals and conformed to the guidelines of the Canadian Council for Animal Care. Experiments at UCL were approved by the UCL Animal Welfare and Ethical Review Body and were carried out in accordance with the Animal (Scientific Procedures) Act (Home Office, UK, 1986) under project license number 70/9098.

METHOD DETAILS

Slice preparation

Slices of the first and second lumbar spinal cord segments (L1 and L2) were prepared for electrophysiology from P7-P14 mice. Mice were anesthetized by intraperitoneal injection of a ketamine (60mg/kg) and xylazine (12mg/kg) mixture. After loss of their righting reflex, mice were cooled on ice and decapitated. Thoracolumbar spinal cords were dissected in an ice-cold oxygenated sucrose solution (in mM: KCL, 3; $NaHCO_3$, 25; KH_2PO_4, 1.2; $MgS0_4$, 1.3; $CaCl_2$, 1.2; glucose, 10; sucrose, 212.5; $MgCl_2$, 2; pH7.4), embedded in low-melting point agarose (Sigma-Aldrich Cat# A4018) and sectioned transversely at 300 μms on a vibratome (Leica VT1200S, Leica). For experiments involving ventral root stimulation, the spinal cord was glued to an agar block and slices were cut at a 45 degrees angle, in order to preserve MN axons, and thickness was increased to 400 μm, to maximize connectivity. For paired recordings, slices were cut on the transverse plane. The dorsal horn ablated preparation was obtained by gluing the spinal cord ventral side up and positioning the blade at the level of the central canal. In these experiments, the cutting solution contained (in mM) 130 K-gluconate, 15 KCl, 0.05 EGTA, 20 HEPES, 25 d-glucose, 3 kynurenic acid and pH 7.4, mimicking the intracellular content of the cells (Bhumbra and Beato, 2018). Slices were then incubated in an oxygenated Ringer's solution (in mM: NaCl, 111; KCl, 3.1; glucose, 11; NaHCO3, 25; MgSO4, 1.25; CaCl2, 2.5; KH2PO4, 1.8; pH 7.4) at room temperature for 1 hour before recording.

Whole-cell patch-clamp recordings and stimulation

Slices were transferred to a recording chamber mounted on a Zeiss AxioExaminer microscope and perfused with oxygenated (95% O2/ 5% CO2) room temperature Ringer's solution. Cells were visualized using a 20x wide aperture (1.2nA) water-immersion objective lens, a CCD camera (CoolSNaction potential EZ, Photometrics, Arizona, USA) and Slidebook 6.0 software (Intelligent Imaging Innovations, Colorado, USA).

Whole cell patch-clamp recordings were made under voltage- and current-clamp (VC and IC, respectively) configurations using a Multiclamp 700B amplifier (Molecular Devices, California, USA). Recordings were low pass filtered at 3 kHz (VC) or 10kHz (IC), and acquired at 25 kHz with CED Power 1401 AD board and Signal software (Cambridge Electronic Design, Cambridge UK). Recording pipettes were filled with a solution containing in mM: K-gluconate, 128; NaCl, 4; $CaCl_2$, 0.0001; HEPES, 10mM, glucose, 1mM; Mg-ATP, 5; and GTP-Li, 0.3, pH 7.2, lucifer yellow dilithium salt (0.4 mg/ml, Thermo Fisher Scientific), and neurobiotin (1mg/ml, Vector Laboratories), and had resistances of 4-6 MΩ. Lucifer yellow allowed for the immediate visualization of the soma and dendrites of the patched cell. This allowed us to avoid photostimulating processes of the recorded cell while stimulating nearby presynaptic cell bodies. Neurobiotin allowed for post hoc confirmation of the identity of the recorded cell and that axons or dendrites of the post-synaptic cell were not stimulated when photostimulating pre-synaptic cell bodies by comparing images of the region of interest (ROIs) stimulated (see below) to the confocal image of the biotin-filled post-synaptic cell (n = 15). Prior to carrying out the photostimulation protocol, rheobase, (defined as the minimal current needed to elicit an action potential 50% of the time, in response to 5 ms depolarizing currents steps) and input resistance (average of 30 responses to a 1 s −10mV hyperpolarizing pulse) were collected to determine cell size and excitability. Ventral roots were stimulated using a suction electrode connected to a Digitimer (UK) constant current stimulator (DS3). Prior to recording from any V3 IN, a MN was recorded in whole cell voltage clamp and the threshold for antidromic activation (when present) or for the occurrence of monosynaptic recurrent excitation was determined. The stimulation intensity for V3 INs recording was then set at 5Xthreshold and kept constant for the duration of the experiment for each slice.

MNI-glutamate perfusion and photostimulation protocol

Photostimulation experiments only proceeded when membrane potential was stable (i.e., did not fluctuate more than 5mv during a 5 minute period), MNI-caged-L-glutamate (2.5mM, Tocris) was perfused in the Ringer's solution at a rate of 2ml/min. Holographic photolysis of MNI-glutamate was performed using a 405 nm laser directed through a Phasor spatial light modulator (SLM) system (Intelligent Imaging Innovations, Colorado, USA), stimulating regions of interest (ROI) as controlled by Slidebook software (Intelligent Imaging Innovations, Colorado, USA). Prior to photostimulating ROIs, direct photostimulation of the patched cell was performed to determine the duration which elicited a single action potential. Ideal pulse duration was 800-1000 μs for V3 INs and 1000-1500 μs for MNs. If longer durations were necessary, the slice was not used as it was deemed to be unhealthy. ROIs were selected as identified neuronal cell bodies under fluorescence, with the ROI blanketing the soma. Once ROIs in a single focal plane were selected, sequential photostimulation of the ROIs was carried out while recording from the post-synaptic neuron. For each ROI, 5 photostimulation pulses of between 800 μs – 1500 μs each (depending on excitability of the slice, as determined by direct photostimulation of the recorded neuron) at 1Hz were delivered. Laser onset and duration were recorded as a waveform for each ROI and collected in Signal along with the recording. Post-synaptic cells were held between −60mv and −50mv for the duration of the experiment (which could exceed one hour). Photomicrographs for each ROI were taken to map the location of each cell relative to the post-synaptic cell recorded.

VC recordings were used to determine EPSC amplitude and onset latency, which was measured as the time from laser onset to the start of the inward current recorded. Rise time was measured as the 10 – 90% of the evoked EPSCs. Five photostimulation pulses at 1Hz were delivered, with the average response size and latency used for analysis. Jitter was quantified as the variance in latencies across the five pulses. In stable preparations, once a connection was established, a second electrode was used to record from the connected pre-synaptic cell to determine if the connection was bi-directional. As the connected cells were often in close proximity

(100-200 μm), the gigaseal of the patch-clamp was often lost on one of the two cells, but loose patch recordings were obtained, confirmed by recording responses from direct photostimulation of the cell body.

Dextran labeling

To determine if the V3 INs that formed connections with ipsi-MNs were also commissural INs, a small unilateral incision was made at the L3 segment in a subset of spinal cords. Dextran crystals (3000mW, ThermoFisher Scienitific) were then applied to the cut cord as previously described (Birinyi et al., 2003; Blacklaws et al., 2015) and the cord was incubated in aCSF for 1.5 to 2 hours. The L2 region was then sectioned and incubated as described above. MNs contralateral to the cut side were then patched while dextran-positive V3 INs ipsilateral to the patched MN were photostimulated, thus allowing to determine if contralateral-projecting V3 INs also formed connections with ipsi-MNs.

Carbenoxolone (CBX) perfusion

In a subset of experiments in which a connection between a $V3_{VLat}$ and ipsi-MN was established, CBX (100 μM, Sigma-Aldrich) was added to the MNI-glutamate aCSF perfusate. VC responses before and during 20 minutes CBX perfusion were compared.

Immunofluorescence and imaging

Upon completion of the electrophysiological recordings, slices were incubated in 4% paraformaldehyde for 1 hour at room temperature followed by three, 15 minute washes in 0.1% PBS-T. Slices were incubated at 4°C overnight in rabbit anti-DsRed primary antibody (Clontech, 1:1000) and in some slices mouse anti-ChAT primary antibody (Millipore, 1:200) followed by a four-hour incubation period in Goat anti-rabbit 594 (Jackson ImmunoResearch Laboratories, 1:500) and Alexa Fluor 647- conjugated streptavidin (ThermoFisher Scientific, 1:500). Images were obtained using a Zeiss LSM 510 upright confocal microscope. Images were compared to photos obtained during the electrophysiology recordings in Slidebook to confirm that the ROIs did not overlap with visually identifiable dendrites or axons.

QUANTIFICATION AND STATISTICAL ANALYSIS

All data are presented as mean ± SD. Sigma-Plot (version 14.0, Systat software, California, USA) was used for all analysis including testing for normality. Un-paired Student's t tests were used for all comparisons except for the carbenoxolone results in which a pairwise t test was used. Statistical significance was set at $p < 0.05$.

DATA AND SOFTWARE AVAILABILITY

All data are available upon request.

Cell Reports, Volume *25*

Supplemental Information

Sub-populations of Spinal V3 Interneurons Form Focal Modules of Layered Pre-motor Microcircuits

Jeremy W. Chopek, Filipe Nascimento, Marco Beato, Robert M. Brownstone, and Ying Zhang

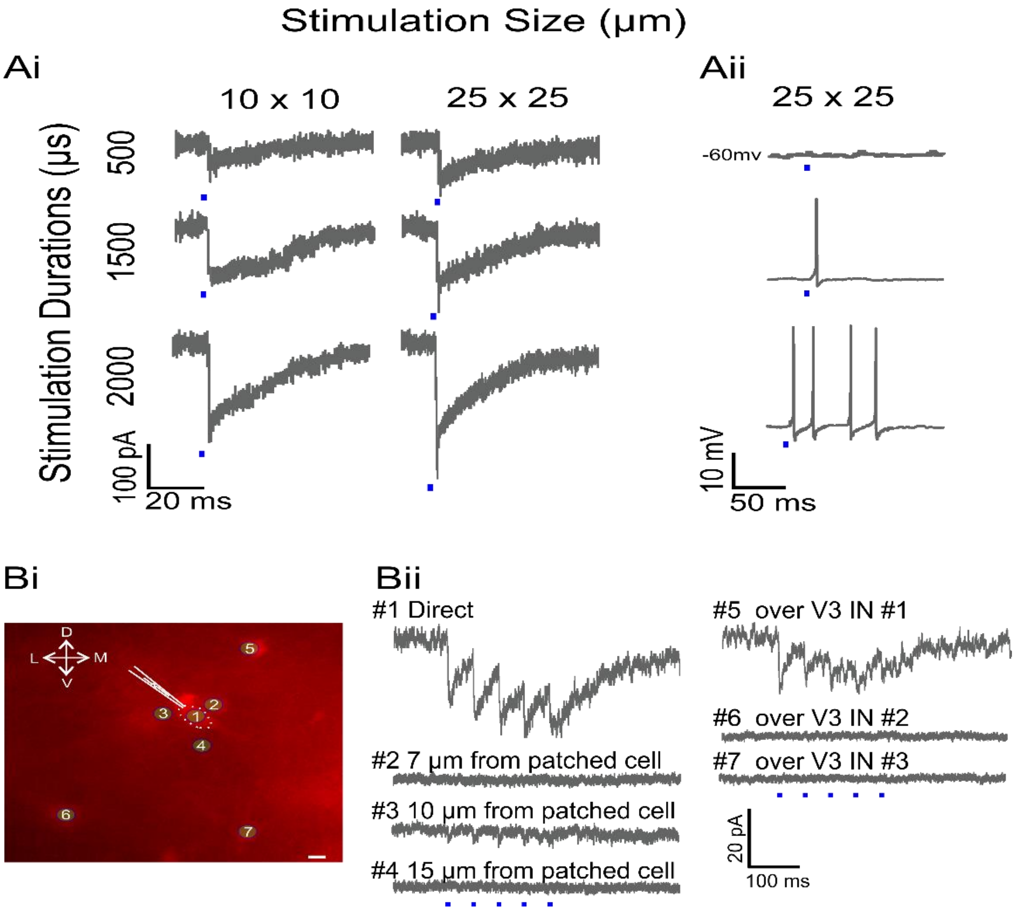

Figure S1. Sensitivity and specificity of photostimulation. Related to Figure 1.

A. Sensitivity of photostimulation. (Ai) Voltage clamp responses to different photostimulation areas (10 x 10 µm and 25 x 25 µm squares) and durations (1000, 1500, 2000 µs) directly over a patched motoneuron. (Aii) Current clamp responses to different durations of photostimulation. **B.** Specificity of photostimulation. (Bi) Spots for photostimulation were placed over the patched V3 IN soma (1), near the patched neuron (2, 3, 4) and on three identifiable pre-synaptic V3 IN somata (5, 6, 7). Scale bar = 10 µm. (Bii) Voltage-clamp recordings showing that the recorded V3 IN responded to direct but not adjacent stimulation as well as to stimulation of 1 of the 3 other V3 INs, demonstrating specificity of stimulation.

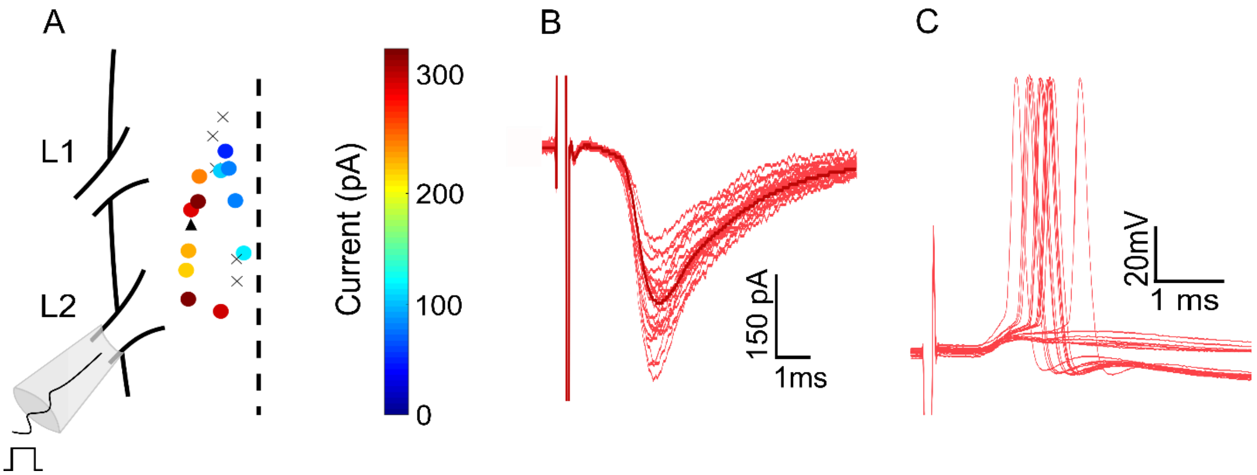

Figure S2. Recurrent excitation of V3 INs by motoneurons in the dorsal horn removed preparation. Related to Figure 4.

(A) Dorsal horn removed preparation in which a suction electrode was used to stimulate the L2 ventral root while recording V3 INs. V3 IN locations are depicted as circles (responded to ventral root stimulation) and X's (did not respond to ventral root stimulation) with colour indicating the strength of the evoked EPSC. Dashed line indicates the central canal. Black arrowhead indicates the location of the V3 IN described in panel B & C. (B) Individual voltage-clamp responses recorded in a patched $V3_{VLat}$ IN in response to L2 ventral root stimulation (30 pulses). Note stimulus artefact has been truncated and subtracted (by fitting with a double exponential). Thick red line indicates average evoked response. (C) Individual current-clamp responses recorded in the same patched V3Vlat IN as in (B) in response to L2 ventral root stimulation.

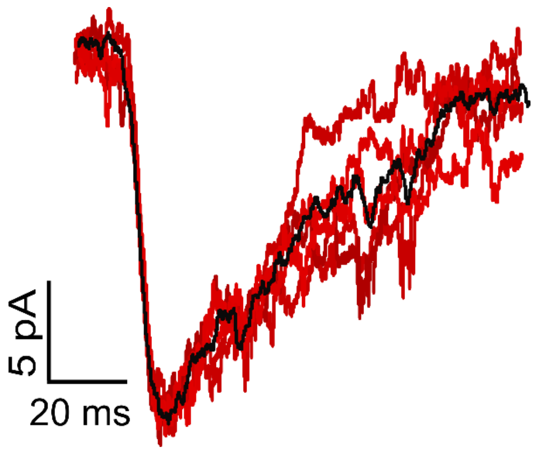

Figure S3. Low jitter of V3$_{VMed}$ IN-evoked EPSCs in V3$_{VLat}$ INs. Related to Figure 5. Responses in a V3$_{VLat}$ IN to 5 consecutive stimulation pulses at 1Hz to a V3$_{VMed}$ IN (red shades), with the average overlaid (black).
